# Spatiotemporal immune heterogeneity and precision immunotherapy for ovarian cancer

**DOI:** 10.1002/ctm2.70836

**Published:** 2026-09-14

**Authors:** Xinfeng Yang, Yida Wang, Haiyue You, Jingyi Gao, Yangkun Feng, Feng Zhang, Yan Zhang, Bing Zhang, Shaojie Zhao, Yanfang Gu

**Affiliations:** ^1^ Department of Oncology Wuxi Maternity and Child Health Care Hospital Women's Hospital of Jiangnan University Jiangnan University Jiangsu China; ^2^ Department of Oncology Wuxi Maternal and Child Health Care Hospital Wuxi Medical Center Nanjing Medical University Wuxi China; ^3^ Suzhou Medical College Soochow University Suzhou Jiangsu China

**Keywords:** combination therapy, immune evasion, immunotherapy, ovarian cancer, precision therapy, spatiotemporal heterogeneity, tumour immune microenvironment

## Abstract

Background: The clinical benefit of immunotherapy in ovarian cancer (OC) remains limited. The primary reason is not simply a lack of immune activation, but rather the marked spatiotemporal heterogeneity of the tumour immune microenvironment. Previous reviews have mainly focused on individual immune components, immunosuppressive mechanisms, or therapeutic approaches, with less systematic integration of distinct anatomical niches, treatment‐stage evolution, and their implications for precision immunotherapy selection.

Main body: In this review, we propose a spatiotemporal immune heterogeneity framework to understand differential immunotherapy responses in OC. We first summarize the distinct immune landscapes across different anatomical niches, highlighting how anatomical niches shape immune suppression, immune exclusion and therapeutic vulnerability. We further discuss temporal remodelling of the tumour immune microenvironment during disease progression, emphasizing that immune states represent dynamic biological processes rather than fixed tumour characteristics. Based on this concept, we review biomarker‐guided stratification strategies. Precision therapeutic strategies are further discussed according to distinct immune ecosystems. Finally, we highlight major challenges in clinical translation, including biomarker standardization, multicentre validation and the development of artificial intelligence‐assisted decision systems.

Conclusion: By linking immune ecosystem evolution, this review provides a framework for transitioning OC immunotherapy towards dynamic and precision‐guided clinical management.

## Highlights

- Ovarian cancer is not uniformly immune‐cold tumour. Its immune states dynamically vary across anatomical sites and treatment stages.
- Precision immunotherapy requires integration of genomic, immune, spatial and circulating biomarkers rather than reliance on single markers.
- Future translation depends on biomarker standardization, multicentre validation and artificial intelligence‐assisted dynamic treatment decision frameworks.

## Graphical abstract

Graphical Highlights

Spatiotemporal immune heterogeneity shapes distinct and evolving immune ecosystems in ovarian cancer. Multimodal biomarkers enable biologically informed patient stratification beyond static single‐marker approaches.

Precision immunotherapy should match dominant immune barriers across lesions and treatment stages. Clinical translation requires standardized assays, prospective validation, and adaptive decision frameworks.

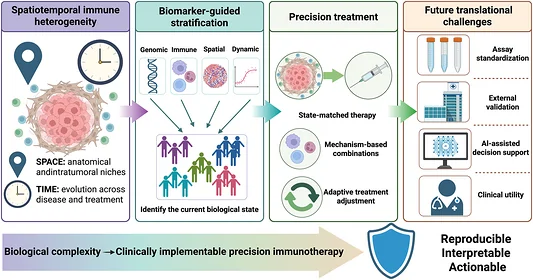

## 1 INTRODUCTION

Ovarian cancer (OC) is the second most common gynaecologic malignancy after endometrial cancer and remains one of the deadliest.^1^ Early symptoms are often nonspecific, and effective population screening is not available, so many patients present with advanced‐stage disease and curative alternatives are limited.^2^ Contemporary Surveillance, Epidemiology and End Results Programme data estimate a 5‐year relative survival of 52.0% for OCs diagnosed in the United States during 2016–2022.^3^ Advanced disease is characterized by diffuse peritoneal dissemination, frequent relapse after platinum‐based therapy and the eventual emergence of drug‐resistant clones.^4^ These clinical features make durable disease control difficult even when initial cytoreduction and chemotherapy are effective.

Immune checkpoint blockade (ICB), particularly programmed cell death protein 1/programmed death‐ligand 1 (PD‐1/PD‐L1)‐directed therapy, has transformed several solid tumours but has shown modest activity in OC.^5^, ^6^, ^7^ In two randomized phase III trials, JAVELIN Ovarian 100 in the first‐line epithelial ovarian cancer (EOC) setting and JAVELIN Ovarian 200 in the platinum‐resistant setting neither achieved the prespecified efficacy endpoints.^8^, ^9^ In the randomized phase III NINJA trial, nivolumab did not improve overall survival (OS), yielded shorter progression‐free survival (PFS) than gemcitabine or pegylated liposomal doxorubicin (PLD), and reached an objective response rate (ORR) of 7.6% versus 13.2% in the control arm.^10^ These findings indicate that ICB is insufficient for most patients with OC.

The tumour microenvironment (TME) encompasses the entire tumour‐associated ecosystem, including tumour, immune, stromal, vascular, extracellular matrix (ECM) and metabolic components. Within the TME, the tumour immune microenvironment (TIME) specifically refers to the immune‐related cellular, molecular, and spatial architecture, comprising immune cells and their interactions with tumour and stromal elements. Based on the degree and activation status of immune infiltration, tumours are commonly classified as immune‐hot characterized by abundant T cell infiltration but often with suppressed function, or immune‐cold marked by a paucity of immune cells and poor response to immunotherapy.^11^ The traditional view simply categorizes OC as an ‘immune‐cold’ tumour and attributes its limited response to immunotherapy solely to the presence of an immunosuppressive TIME. It is no longer sufficient to explain the modest activity of its immunotherapy. Immunosuppression is undoubtedly important, but the TIME of OC changes with anatomical location and treatment stage. For example, single‐cell sequencing and spatial transcriptomic studies have shown that primary tumours of high‐grade serous ovarian cancer (HGSOC) typically exhibit a more active immune microenvironment than omental metastases, which are more prone to forming an immunosuppressive niche.^12^

**FIGURE 1:**
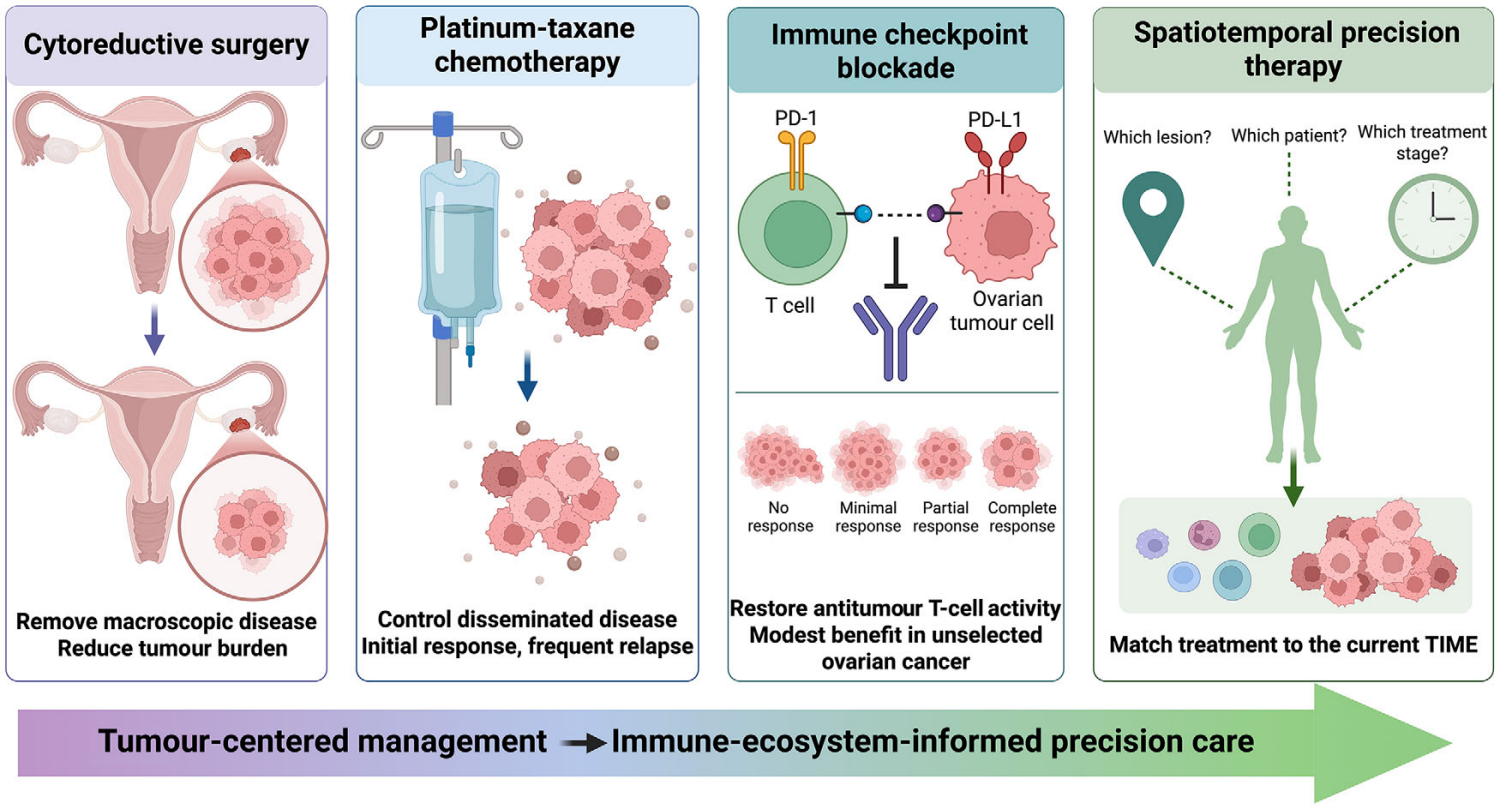
Evolution of ovarian cancer treatment. Ovarian cancer treatment has evolved from cytoreductive surgery and chemotherapy to immune checkpoint blockade and, ultimately, spatiotemporal immune heterogeneity‐guided precision therapy tailored to the patient, lesion and treatment stage.

Therefore, the key question in OC immunotherapy is not simply ‘whether to use immunotherapy’, but rather which immunotherapeutic strategy is biologically most appropriate for a specific patient, a specific lesion, and a specific treatment stage (Figure 1). Based on this understanding, it is necessary to analyse the OC immune ecosystem from a spatiotemporal evolution perspective, providing guidance for precision therapy in OC.

## 2 SPATIOTEMPORAL IMMUNE HETEROGENEITY IN OVARIAN CANCER

The TIME in OC varies across both anatomy and disease course. Primary tumours, invasive margins, peritoneal and omental metastases, ascites, and recurrent lesions differ in immune‐cell composition, stromal organization, and suppressive signalling, and treatment continuously reshapes these states. It is necessary to systematically understand the spatiotemporal dynamics of the immune microenvironment in OC.

### 2.1 Anatomical site‐specific immune features

OC disseminates predominantly within the peritoneal cavity. Free‐floating tumour cells, primary lesions, peritoneal and omental metastases, malignant ascites, and their surrounding stromal and immune compartments form an interconnected but spatially heterogeneous ecosystem. This heterogeneity involves not only differences in immune‐cell composition and functional state, but also variation in cell‐matrix organization, vascular structure, metabolic conditions and the regional distribution of intercellular signalling networks. These spatial features have been associated with disease progression, chemotherapy response, and clinical outcome and may also influence sensitivity to immunotherapy.

The immune landscape differs across anatomical sites. Recent paired analyses showed that primary HGSOC lesions generally exhibited a more immunologically active microenvironment than matched omental metastases, although the magnitude and composition varied among patients.^12^ Peritoneal and omental metastases are exposed to soluble mediators, nutrients, lipids, and hypoxic stress within the peritoneal fluid and are further shaped by mesothelial cells, adipocytes, fibroblasts and tissue‐resident myeloid populations. These metastatic niches are frequently enriched in immunoregulatory macrophages and other suppressive myeloid cells, together with impaired antigen‐presentation and interferon‐response programmes.^13^ In a study of 24 paired, chemotherapy‐naïve primary and metastatic HGSOC samples, immune‐effector‐cell infiltration in metastatic lesions did not consistently translate into improved outcomes.^14^ Consistent with this observation, a retrospective immunohistochemical analysis of omental metastases from 100 patients with advanced EOC found that high cluster of differentiation 68‐positive macrophage associated with poorer survival.^15^ These findings suggest that site‐specific immune organization may partly account for heterogeneous responses among lesions within the same patient. They also support multisite sampling and site‐aware therapeutic design.

Malignant ascites represents a distinct ‘liquid niche’ of OC dissemination rather than merely a clinical consequence of advanced disease. Ascites commonly contains suppressive myeloid populations, dysfunctional T and natural killer (NK) cells, cytokines, tumour cells, chemokines, extracellular vesicles (EVs), metabolites and damage‐associated molecular patterns. Their relative abundance and functional effects vary according to histotype, disease burden, treatment exposure and sampling time.^16^ Functional studies using patient‐derived ascites have shown that it can inhibit NK cell activation, degranulation, cytokine production, and tumour‐cell killing. This suppression has been linked to several soluble and metabolic factors, including cancer antigen 125 (CA‐125), transforming growth factor β (TGF‐β), electrolyte dysregulation, and lipid accumulation.^17^, ^18^, ^19^ In a clinical–translational study of patients with advanced EOC, stronger ascites‐mediated NK cell inhibition and higher peritoneal TGF‐β levels were associated with shorter PFS and OS.^18^ Thus, the ascitic compartment constitutes an active immunometabolic environment that may simultaneously impair antitumour immunity and facilitate tumour‐cell survival, adhesion, implantation, and peritoneal dissemination.

### 2.2 Immune localization and mechanisms of immune exclusion revealed by spatial‐transcriptomics and single‐cell sequencing

If inter‐site variation represents the macroscopic dimension of spatial heterogeneity in OC, the integration of spatial‐transcriptomics with single‐cell sequencing has begun to reveal its microscopic basis by resolving the intratumoral localization of immune cells and the molecular programmes associated with immune exclusion. In many tumours, immune cells are present but are unevenly distributed, preferentially accumulating at the invasive margin or within stromal compartments while remaining largely excluded from tumour‐cell‐rich regions.^20^ This spatial segregation may limit productive interactions between immune and tumour cells and contribute to a mismatch between immune‐cell presence and effective antitumour activity.

Integrated spatial and single‐cell analyses have identified a relatively consistent gradient between the tumour core and invasive margin in several OC cohorts, involving immune‐cell density, effector programmes, and immune‐checkpoint expression. Tumour‐core regions are frequently associated with enhanced fibroblast activation and transcriptional programmes related to ECM deposition and remodelling. By contrast, invasive margins may show greater enrichment of antigen‐presentation, interferon‐response, and cytotoxicity‐associated signatures.^21^ These observations support a spatially layered model in which immune activation is relatively preserved at the tumour periphery but progressively constrained toward the tumour core. Importantly, this pattern is unlikely to arise from a single dominant mechanism. Rather, immune exclusion probably reflects the combined effects of ECM‐mediated physical restriction, abnormal vasculature, suppressive stromal programmes, inhibitory receptor‐ligand signalling, altered chemokine gradients and metabolic competition.

Within this spatially organized microenvironment, predicted intercellular communication networks also appear to differ between anatomical regions. In tumour‐core areas, tumour cells, myeloid populations, and stromal cells may preferentially express ligand‐receptor programmes associated with immunosuppression, tissue repair, chemotaxis, and nutrient competition. These inferred networks may restrict T cell infiltration and impair effector function. At the invasive margin, interactions involving dendritic cells (DCs) and T cells are more frequently associated with antigen presentation, interferon signalling, and lymphocyte activation, suggesting that peripheral regions may be more permissive to the initiation or maintenance of local antitumour immune responses.^21^

Notably, many commonly used spatial‐transcriptomics platforms currently operate at spot‐level rather than single‐cell resolution, and each spot may contain mixtures of tumour, immune, endothelial and stromal cells. Cell–cell interactions inferred by integrating spatial‐transcriptomic and single‐cell data still require further validation through multiplex immunofluorescence (IF), immunohistochemistry (IHC), spatial proximity analysis and functional experiments.

Despite these methodological limitations, these spatial omics‐based findings help us understand why, even when immune cells are present in certain regions of the same lesion, immunotherapy often fails to mount an effective antitumour response within the tumour core, which remains densely populated by malignant cells.

### 2.3 Tertiary lymphoid structures as spatial immune‐activation niches

In some OC lesions, highly organized tertiary lymphoid structures (TLSs) can be found. These are ectopic lymphoid formations that develop in non‐lymphoid tissues under conditions of chronic inflammation or persistent tumour antigen stimulation. Unlike immune‐excluded regions, the TLS‐enriched local niche may facilitate antigen presentation, T cell and B cell cooperation, clonal expansion of lymphocytes, and sustained local immune activation.

The functional relevance of TLSs is closely related to their maturation status. Early or immature TLSs may appear only as focal aggregates of B cells, T cells, and myeloid cells, whereas mature TLSs show more clearly organized B‐cell follicles, germinal centre‐like structures, follicular dendritic‐cell networks, and T/B cell compartmentalization. At the same time, DCs can locally present tumour antigens and activate cluster of differentiation 8 (CD8)^+^ T cells, while T follicular helper cells support B cell differentiation and antibody responses. TLSs can therefore function as local secondary lymphoid organ‐like platforms in tumour tissue, spatially coupling antitumour humoral and cellular immunity.^22^

TLS‐rich areas may provide spatial niches that support antigen presentation, T cell‐B cell cooperation, and local adaptive immune activation. It should be considered an important and biologically distinct component of spatial immune heterogeneity in OC.

### 2.4 Temporal immune evolution from premalignant disease to first‐line treatment

In addition to spatial heterogeneity, the TIME undergoes continuous remodeling during disease development, progression and therapeutic exposure. The same patient may exhibit substantially different immune states at different stages of the disease course. Importantly, the evidence supporting temporal immune heterogeneity in OC does not derive exclusively from truly longitudinal sampling. A considerable proportion of the proposed ‘immune evolution’ has instead been reconstructed by comparing human tissues obtained at different pathological stages.

Temporal heterogeneity may emerge before clinically overt disease develops. Kader et al. analysed 44 fallopian‐tube specimens from 43 individuals collected across multiple centres and integrated high‐throughput cyclic IF imaging with spatial‐transcriptomics to reconstruct the progression from normal fallopian tube epithelium and tumour protein p53 signatures to serous tubal intraepithelial carcinoma (STIC) and invasive HGSOC. Interferon‐related signalling, tissue‐resident memory T cells, and NK‐conventional type 1 DC‐cytotoxic T lymphocyte‐associated immune surveillance were already detectable in tumour protein p53 signatures and early STIC lesions. With progression toward more advanced STIC, NK and conventional type 1 DC‐associated surveillance became less prominent, whereas regulatory T cells (Tregs) and suppressive antigen‐presenting cells increased, along with exhausted CD8^+^ T cells and lymphocyte‐activation gene 3 (LAG‐3)^+^cluster of differentiation 4 (CD4)^+^ T cells.^23^ These findings suggest that early HGSOC development may not represent a simple transition from an immunologically silent state to immune suppression, but rather a progressive process involving immune recognition, immune selection, and eventual escape.

Once disease progresses to clinically apparent advanced HGSOC, treatment itself becomes a major driver of TIME evolution. The clinical trajectory is not uniform. Some patients undergo primary debulking surgery followed by adjuvant platinum‐taxane chemotherapy, whereas others receive neoadjuvant chemotherapy (NACT), followed by interval debulking surgery (IDS) and additional systemic treatment.^24^

Within this temporal framework, surgery represents a distinct immunological transition point. Cytoreduction rapidly decreases tumour burden and may reduce persistent antigenic stimulation and the production of tumour‐derived immunosuppressive mediators. At the same time, extensive tissue injury, ischemia‐reperfusion, and wound repair induce acute inflammatory responses, mobilization of neutrophils and monocytes, and the release of immune‐regulatory cytokines. Surgery should not be classified simply as either immunostimulatory or immunosuppressive, but rather as a profound and time‐dependent immune reset.

Clinical studies illustrate this complexity. Napoletano et al. compared 25 patients with treatment‐naïve OC, 25 patients undergoing IDS after NACT, 25 patients with recurrent OC, and 25 patients with benign gynaecological disease. Peripheral blood was collected before surgery, 2–4 days after surgery, and approximately 15 days postoperatively. Following primary cytoreductive surgery, circulating CD4^+^cluster of differentiation 25 (CD25)^+^ forkhead box P3^+^ Tregs rapidly decreased, serum interleukin‐10 (IL‐10) levels declined, and CD8^+^ T cell function improved. In contrast, these changes were less evident in patients who had already received NACT before IDS and in patients undergoing surgery for recurrent disease.^25^ These findings indicate that the immunological consequences of surgery rely substantially on prior treatment exposure and disease stage.

Longitudinal clinical evidence indicates that immune remodelling during first‐line therapy is not unidirectional. In one study of 90 patients with newly diagnosed OC, peripheral blood was collected sequentially at different stages of initial treatment. For patients who received NACT followed by IDS. Several circulating immunosuppressive parameters decreased after NACT, suggesting that early chemotherapy exposure may create a transiently more favourable systemic immune state. Surgery subsequently altered this profile again, with some suppressive parameters rebounding, while completion of first‐line treatment was associated with another broader shift in immune‐related circulating proteins.^26^

Longitudinal analyses of tumour tissue provide further evidence that chemotherapy can enhance selected immune responses while simultaneously establishing new suppressive niches. Launonen et al. analysed 117 pre‐ and post‐chemotherapy HGSOC specimens using single‐cell RNA sequencing, cyclic multiplex IF, bulk RNA sequencing, and spatial‐transcriptomics, with paired pre‐treatment and IDS samples available for a subset of patients. Chemotherapy markedly remodelled myeloid cell states and was associated with the formation of spatially organized multicellular myeloid structures termed ‘Myelonets’. These structures were associated with CD8^+^ T cell exhaustion and exclusion. They were more prominent in tumours with poor chemotherapy responses. Single‐cell and spatial analyses further identified nectin cell adhesion molecule 2‐T‐cell immunoreceptor with immunoglobulin and ITIM domains (TIGIT) as one of the candidate inhibitory interactions between chemotherapy‐remodelled myeloid cells and CD8^+^ T cells. Importantly, the study extended beyond ligand‐receptor inference by establishing immune‐competent ex vivo cultures from 11 patients; combined or individual blockade of TIGIT and PD‐1 enhanced CD8^+^ T cell effector activity in a subset of samples, providing additional functional support for the biological relevance of this pathway.^27^

### 2.5 Maintenance therapy and recurrence: Immune resetting

Following completion of initial surgery and chemotherapy, maintenance treatments such as anti‐angiogenic agents and poly(ADP‐ribose) polymerase inhibitors (PARPis) continue to impose selective pressure on the TIME.

Anti‐angiogenic therapy represents an important factor that can reshape the OC immune microenvironment. Excessive vascular endothelial growth factor (VEGF) signalling enhances abnormal vascular architecture, increased vascular permeability and impaired immune‐cell trafficking. VEGF blockade may induce transient vascular normalization, improve vascular integrity and immune‐cell accessibility.^28^ In the GEICO‐89T/MINOVA study, addition of bevacizumab to NACT was associated with increased CD8^+^ T cell infiltration and decreased CD4^+^ T cell populations.^29^

For poly(ADP‐ribose) polymerase (PARP) inhibition, a single‐centre phase II study evaluated olaparib combined with the anti‐PD‐L1 antibody durvalumab in 35 patients with recurrent OC. Treatment was associated with increases in interferon γ (IFN‐γ), C‐X‐C motif chemokine ligand 9/10, tumour‐infiltrating lymphocytes, and, in some samples, PD‐L1 expression, suggesting the emergence of an immunostimulatory state during therapy.^30^ These data support the capacity of PARP inhibition to remodel the TIME.

With prolonged therapeutic selection and clonal evolution, recurrence represents another major transition in temporal immune evolution. A paired analysis of primary and recurrent tumours from nine patients with advanced HGSOC, using multiplex IHC/IF and NanoString profiling, with validation in an independent RNA‐sequencing cohort containing 66 paired primary and recurrent samples, showed that many recurrent lesions contained increased T cell and myeloid cell infiltration. Recurrence was also associated with increased PD‐L1, stromal signalling, and expression of inhibitory receptors including TIGIT and cytotoxic T‐lymphocyte‐associated protein 4 (CTLA‐4).^31^ These findings argue some recurrent tumours may remain highly infiltrated while simultaneously developing stronger adaptive immune resistance.

**FIGURE 2:**
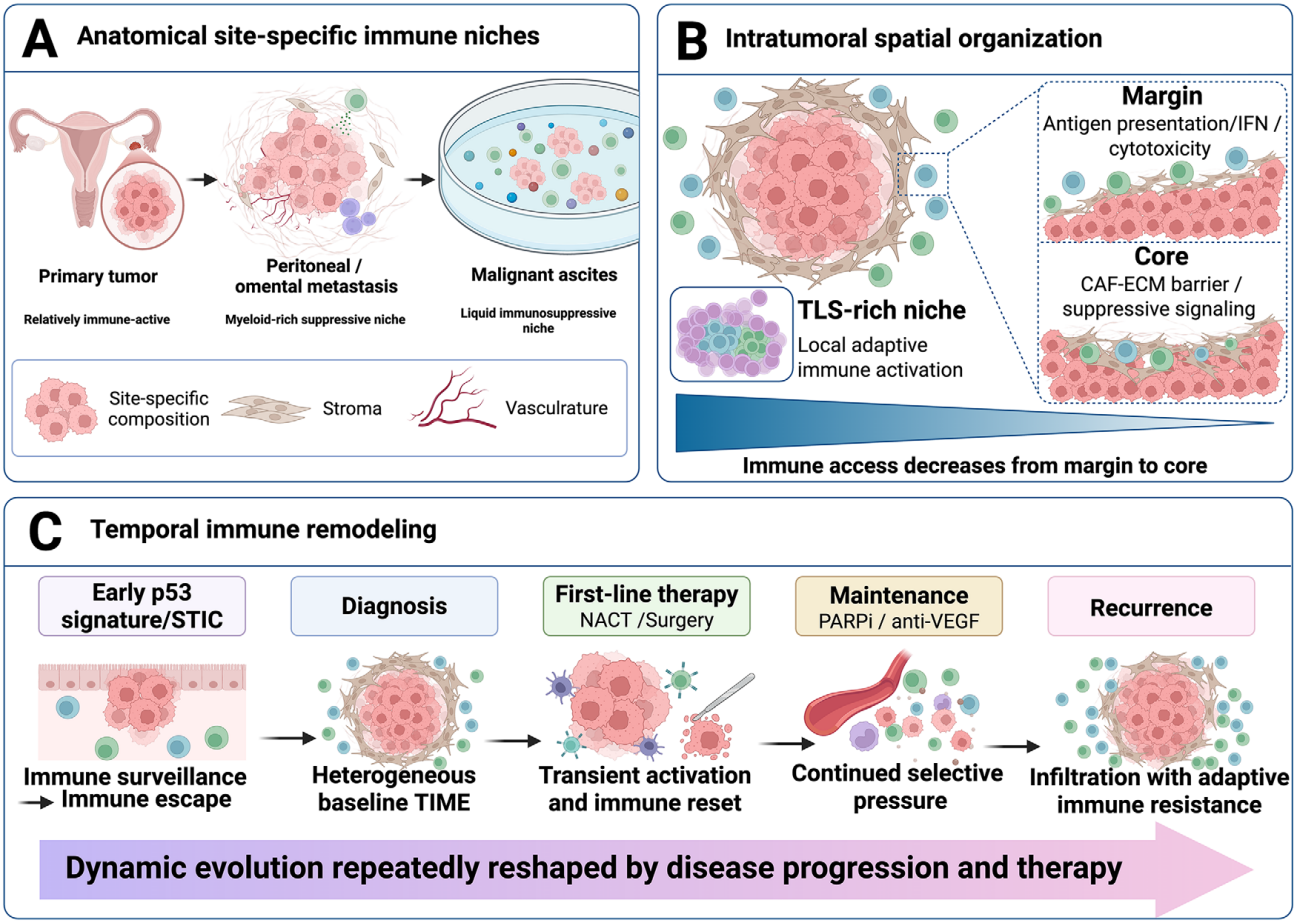
Spatiotemporal immune heterogeneity in ovarian cancer. Immune ecosystems differ across anatomical sites and intratumoral niches and are dynamically remodelled during tumour evolution, treatment and recurrence.

Overall, temporal immune evolution in OC is not a unidirectional progression from immune activation to immune suppression, but rather a dynamic process that is repeatedly reshaped by disease progression and therapeutic pressure. Immunotherapy in OC should identify the patient's current immune evolutionary stage and select the corresponding treatment strategy based on the specific TIME state (Figure 2, Tables 1 and 2).

**TABLE 1:** Spatial heterogeneity of the ovarian cancer immune microenvironment.

Spatial compartment	Key immune features	Functional significance	References
Primary lesion	Variable immune activity; often more active than matched omental metastases; mixed CD8^+^ T/NK/APC and suppressive myeloid/stromal cells.	Inflamed, excluded and desert states may coexist; primary tissue is not a patient‐wide surrogate.	^12^, ^20^, ^21^
Peritoneal/omental metastases	Suppressive myeloid cells, fibroblasts, adipocytes, and mesothelium; weaker antigen‐presentation/IFN programmes.	Stromal, lipid and myeloid signals promote immune dysfunction and exclusion.	^12^, ^14^, ^15^, ^16^
Malignant ascites	Tumour cells, suppressive myeloid cells, dysfunctional T/NK cells, cytokines, EVs, and metabolites.	Dynamic liquid niche linked to peritoneal lesions, but not a substitute for solid tissue.	^13^, ^16^, ^17^, ^18^, ^19^
TLS‐rich niche	Organized B cells, T cells, dendritic cells, and plasma cells with local antigen presentation.	Supports local priming and T‐B cooperation; significance depends on location and maturation.	^22^

Abbreviations: APC, antigen‐presenting cell; EV, extracellular vesicle; IFN, interferon; TLS, tertiary lymphoid structure.

**TABLE 2:** Temporal immune remodelling across the ovarian cancer treatment trajectory.

Disease/treatment stage	Key immune remodelling	Functional significance	References
Early precursor	Early immune surveillance may shift toward Treg accumulation, suppressive APCs, and T cell dysfunction.	Immune escape can begin before disseminated disease.	^23^
Advanced, treatment‐naive diagnosis	Lesions span inflamed, excluded, and desert states with marked site‐to‐site variation.	Diagnostic specimens represent only the state at a specific time point and anatomical location, not a fixed patient trait.	^12^, ^14^, ^15^, ^20^, ^21^, ^22^
Early neoadjuvant chemotherapy	Immunogenic cell death and DAMP release may transiently increase inflammation.	Chemotherapy may create a temporary immune‐responsive window.	^26^, ^27^, ^29^
Primary/interval debulking surgery	Tumour removal, perioperative inflammation, wound healing, and immune‐cell redistribution.	A time‐dependent immune reset.	^25^, ^26^
Completion of chemotherapy	Persistent myeloid/neutrophil programmes, Myelonets, CD8^+^ exhaustion/exclusion.	Initial activation may evolve into adaptive and myeloid‐dominant resistance.	^27^
Maintenance	Vascular normalization or IFN/CXCL9‐CXCL10 activation.	Maintenance can improve access while imposing new selective pressures.	^28^, ^29^, ^30^
Recurrent	T/myeloid infiltration may coexist with PD‐L1, TIGIT, and CTLA‐4.	Recurrent tumours can appear immune‐infiltrated but establish stronger adaptive immune resistance.	^31^

Abbreviations: CXCL9, C‐X‐C motif chemokine ligand 9; CXCL10, C‐X‐C motif chemokine ligand 10; CTLA‐4, cytotoxic T‐lymphocyte‐associated protein 4; PD‐L1, programmed death‐ligand 1; DAMP, damage‐associated molecular pattern; TIGIT, T‐cell immunoreceptor with immunoglobulin and ITIM domains.

## 3 FUNCTIONAL HETEROGENEITY AND DYNAMIC PLASTICITY OF MAJOR IMMUNE CELLS IN THE OVARIAN CANCER IMMUNE MICROENVIRONMENT

**FIGURE 3:**
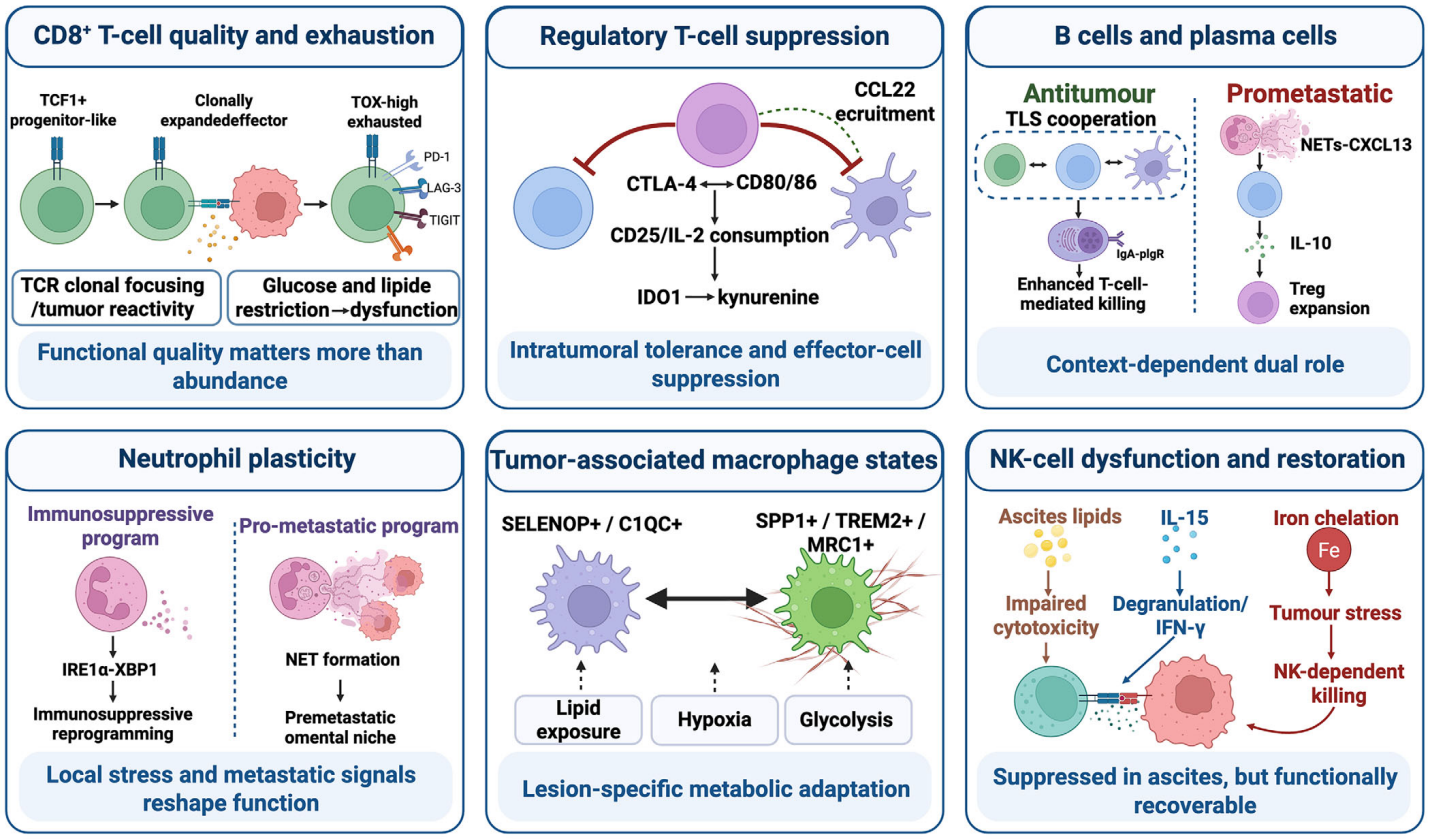
Functional heterogeneity and plasticity of major immune cells. CD8^+^ T cells, regulatory T cells, B/plasma cells, neutrophils, tumour‐associated macrophages and natural killer cells adopt context‐dependent antitumour or immunosuppressive states.

The spatial and temporal heterogeneity described above provides the organizational framework through which the OC immune microenvironment evolves, but these patterns are ultimately generated by the composition, functional states and interactions of individual immune‐cell populations. The same immune‐cell lineage can exert markedly different effects depending on its differentiation state, anatomical location, disease stage and treatment exposure. The following section examines the major immune‐cell populations that constitute the OC microenvironment (Figure 3).

### 3.1 CD8^+^ T cells quality, clonal focusing and exhaustion

CD8^+^ T cells are among the principal effectors of adaptive antitumour immunity in OC. Tumour‐infiltrating CD8^+^ T cells comprise a heterogeneous spectrum of functional states, ranging from relatively less differentiated populations with proliferative potential, through tissue‐resident and clonally expanded effector states, to progressively dysfunctional and terminally exhausted phenotypes.

Within this continuum, T‐cell factor 1 (TCF1) is an important marker distinguishing progenitor‐like exhausted T cells with preserved proliferative capacity from more differentiated exhausted populations. TCF1, encoded by transcription factor 7, is generally associated with a less differentiated state, self‐renewal capacity, and the ability to generate more differentiated effector progeny. By contrast, thymocyte selection‐associated high mobility group box (TOX) contributes to the establishment and maintenance of exhaustion‐associated transcriptional and epigenetic programmes. TCF1 and TOX, however, should not be regarded as simple opposing markers. TCF1^+^ progenitor‐like exhausted cells can sustain the exhausted T cell pool and give rise, under persistent antigen stimulation, to more differentiated TOX‐high cells with reduced proliferative capacity.^32^ In OC, single‐cell and T‐cell receptor (TCR) clonotype analyses have revealed state continuity and clonal relationships among progenitor‐like, effector‐like, and highly exhausted CD8^+^ T cell populations.^33^ However, direct lineage progression from TCF1^+^ progenitor cells to TOX‐high terminally exhausted cells is still supported primarily by evidence from other tumour types.^34^

In addition to differentiation and exhaustion status, the clonal structure of CD8^+^ T cells and their responsiveness to autologous tumour cells represent critical parameters for assessing antitumour immunity in OC. TCR clonal expansion suggests that specific T cell populations have undergone antigen‐driven selection. Autologous tumour recognition and cytokine release assays can serve as functional readouts of their tumour reactivity. In HGSOC patient specimens, a more focused intratumoral TCR repertoire has been associated with longer progression‐free and OS.^35^ This observational evidence supports an association between the emergence of dominant T cell clones and more effective antitumour immunity. Accordingly, integrating TCR clonal architecture, autologous tumour reactivity, and the corresponding functional states may provide a more faithful description of antitumour CD8^+^ T cell immunity in OC.

Local nutrient availability and metabolic conditions also contribute to shaping the functional state of CD8^+^ T cells. Patient‐derived studies have indicated that the OC microenvironment can restrict glucose availability to T cells, thereby reducing CD8^+^ T cell polyfunctionality and survival.^36^ Impaired lipid utilization provides another mechanism: CD8^+^ T cells isolated from malignant ascites exhibit reduced lipid uptake and abnormal membrane localization of fatty acid‐binding protein 5. Fatty acid‐binding protein 5‐dependent lipid uptake and mitochondrial metabolism are disrupted. This also leads to CD8^+^ T cell dysfunction and exhaustion.^37^

### 3.2 Regulatory T cells and adaptive immune suppression

Tregs are essential for maintaining peripheral immune tolerance and constitute an important component of the TIME in OC. A study of 104 patients found that CD4^+^CD25^+^forkhead box P3^+^ Tregs were preferentially enriched in tumour tissue and malignant ascites. These patient‐derived Tregs suppressed tumour‐specific T cell responses, while ovarian tumour cells and microenvironmental macrophages produced C‐C motif chemokine ligand 22, which promoted Treg recruitment.^38^ Greater tumour‐associated Treg accumulation was associated with reduced survival.

More recently, single‐cell RNA sequencing has suggested that Treg accumulation may begin much earlier in HGSOC development than previously appreciated. In a 2025 study of stage I HGSOC, single‐cell profiling of 21 697 cells from two incidentally diagnosed, treatment‐naïve patients showed that Tregs constituted approximately 35% of intratumoral CD4^+^ T cells; their presence was subsequently confirmed by IHC in an additional 11 stage I HGSOC tumours.^39^

Mechanistically, Tregs can suppress antitumour immunity through several complementary routes. CTLA‐4 can reduce CD28‐dependent co‐stimulation by engaging cluster of differentiation 80/86 on antigen‐presenting cells. High CD25 expression enables Tregs to efficiently consume locally available interleukin‐2. Treg‐associated cluster of differentiation 39 and cluster of differentiation 73 can also contribute to extracellular adenosine generation, creating conditions unfavourable for cytotoxic lymphocyte activity.^40^ Although these mechanisms are well established in Treg biology, direct evidence for their operation in human OC lesions is still absent.

The local metabolic environment may further influence the balance between Tregs and T cells in OC. Studies of patient tumour tissues have shown that the tryptophan‐catabolizing enzyme indoleamine 2,3‐dioxygenase 1 is aberrantly expressed in OC. It is associated with disease progression and unfavourable outcomes. Mechanistic studies further indicate that indoleamine 2,3‐dioxygenase 1‐mediated tryptophan depletion and kynurenine accumulation can suppress effector T cell function and may create conditions that favour the maintenance of Treg programmes, although direct evidence for the latter in OC lesions is still lacking.^41^

Collectively, Tregs constitute an appealing target for immunotherapeutic intervention. Signalling pathways involving CTLA‐4, CD25 and related molecules provide biologically rational intervention points; however, the majority remain clinically unvalidated in OC. Moreover, indiscriminate Treg depletion risks disrupting systemic immune tolerance and provoking autoimmune toxicity. A more prudent approach would therefore be to selectively interfere with the recruitment, stability, or suppressive activity of intratumoral Tregs.

### 3.3 The dual role of B cells and plasma cells

Compared with T cells, B cells and plasma cells have historically received less attention in OC immunology, but they are not passive bystanders in the TME. In a study integrating three independent HGSOC cohorts comprising 534 patients, intratumoral cluster of differentiation 19^+^ B cell infiltration was associated with improved OS and positively correlated with T cell infiltration.^42^ Notably, intraepithelial T cells showed a clearer survival advantage only when B cells were also present within tumour nests. Cluster of differentiation 138^+^ plasma cell infiltration was likewise associated with favourable survival in HGSOC.^22^ These patient‐level associations support a contribution of B cells and plasma cells to effective antitumour immunity.

The immunostimulatory role of B cells is particularly well supported by OC data on immunoglobulin A (IgA)‐mediated humoral immunity. In the same study of 534 HGSOC patients, analysis of 29 fresh tumour specimens showed that IgA was the main antibody type produced by plasma cells. Functional experiments further showed that IgA could undergo polymeric immunoglobulin receptor‐dependent transcytosis through OC cells, inducing transcriptional changes that included increased IFN‐γ receptor expression, suppression of ephrin‐related programmes and increased expression of dual‐specificity phosphatases. These changes attenuated mitogen‐activated protein kinase kinase‐extracellular signal‐regulated kinase pathway signalling and enhanced tumour‐cell sensitivity to T‐cell‐mediated killing, which was also supported in human autologous tumour‐infiltrating lymphocyte (TIL)‐tumour cell systems.^42^

Conversely, B cells can acquire immunosuppressive and prometastatic functions in specific microenvironments. A study focused specifically on the immune microenvironment of the omentum before overt omental metastasis develops in OC. The investigators compared histologically normal omental tissues from 10 women without a history of malignancy with those from 10 patients with stage I/II HGSOC. Cluster of differentiation 19^+^cluster of differentiation 34^+^ innate‐like B cells accumulated together with neutrophil extracellular traps (NETs) in the omentum of patients with early OC. Mechanistic experiments showed that NETs induced C‐X‐C motif chemokine ligand 13 expression in the premetastatic omentum, recruited innate‐like B cells from the peritoneal cavity, and promoted their production of IL‐10. These B cells subsequently increased local Treg abundance and facilitated omental metastasis. When wild‐type or IL‐10‐deficient cluster of differentiation 43^+^ B cells were transferred into mice lacking mature B cells, only wild‐type B cells markedly increased the omental Treg/type 1 helper T cell ratio and promoted metastasis, whereas the prometastatic effect was substantially attenuated when IL‐10‐deficient B cells were used.^43^

Simply classifying B cells as either ‘antitumour’ or ‘protumour’ in OC is insufficient. To understand their biological significance, it is necessary to determine whether an immunostimulatory state characterized by antigen presentation and antibody effector functions‐predominates, or whether an immunosuppressive state driven by immunoregulatory programmes such as IL‐10 production dominates.

### 3.4 Functional heterogeneity of neutrophils

Neutrophils are a highly dynamic myeloid population within the OC TIME. Although they contribute to acute inflammatory defence under physiological conditions, persistent exposure to tumour‐derived chemokines, growth factors and metabolic stress can promote phenotypes associated with angiogenesis, immune suppression, and metastatic dissemination.

Endoplasmic reticulum stress represents one mechanism underlying this immunosuppressive reprogramming. A HGSOC study showed that intratumoral neutrophils exhibited stronger inositol‐requiring enzyme 1 α (IRE1α)‐X‐box binding protein 1‐associated stress responses than neutrophils from non‐tumour sites. In a spontaneous HGSOC mouse model, neutrophil‐specific deletion of IRE1α delayed primary tumour growth, enhanced early T‐cell‐mediated tumour control, and prolonged survival. Notably, combining PD‐1 blockade with neutrophil‐specific IRE1α deficiency induced tumour regression and long‐term survival in approximately half of the mice.^44^

Neutrophils can also generate NETs. A 2019 study using orthotopic OC models showed that neutrophils accumulated in the omentum before tumour‐cell arrival and released NETs that contributed to formation of a premetastatic niche; inhibition of NET formation reduced subsequent omental colonization.^45^

Neutrophils in OC are not a fixed immunosuppressive population, but rather a highly plastic compartment whose functions are shaped by local stress signals and metastatic niches.

### 3.5 State heterogeneity and remodelling of tumour‐associated macrophages

Macrophages are abundant and highly plastic components of the OC TIME. The conventional M1/M2 framework provides a useful conceptual description of inflammatory versus tissue‐repair‐associated macrophage functions: M1‐like programmes are generally associated with inflammatory activity and tumour‐cell killing, whereas IL‐10 and VEGF expression, tissue repair, angiogenesis, and extracellular‐matrix remodelling are commonly assigned to M2‐like programmes.^46^ However, this binary classification does not adequately capture the complexity of human OC myeloid populations.

Single‐cell and spatial studies have identified multiple tumour‐associated macrophage (TAM) states characterized by genes such as secreted phosphoprotein 1 (*SPP1*), apolipoprotein E, complement C1q C chain, triggering receptor expressed on myeloid cells 2, and mannose receptor C‐type 1. These populations differ in developmental origin, lipid metabolism, phagocytic capacity, matrix interactions, and anatomical distribution.^47^ A more instructive question is which TAM states continue to drive immunosuppression or metastasis at specific lesions and treatment stages.

Among recently characterized TAM states, *SPP1* ^+^ macrophages have received particular attention. These cells are frequently associated with extracellular‐matrix remodelling, angiogenesis, tissue repair and immune suppression and are often enriched in stromal or hypoxic regions. Notably, a hepatocellular carcinoma study integrating pathological assessment, bulk RNA sequencing, single‐cell transcriptomics, and spatial profiling identified a close spatial association between *SPP1* ^+^ M2‐like macrophages and high‐risk tumour regions enriched in NETs, suggesting that macrophages and neutrophils may cooperate in the formation of prometastatic niches.^48^ However, this mechanistic relationship has been established in hepatocellular carcinoma. Given the extensive myeloid remodelling and NET formation observed in malignant ascites, omental metastases, and peritoneal implants of ovarian ECM cancer, these cross‐cancer findings provide a biologically plausible basis for investigating whether *SPP1* ^+^ TAMs contribute to NET‐enriched metastatic niches through remodelling, chemokine signalling and neutrophil recruitment.

The heterogeneity of macrophages described above is closely linked to the adaptation of TAMs to the local metabolic environment. In a spatial analysis of 102 EOC specimens, omental metastases contained more cluster of differentiation 163^+^ TAMs than lesions arising in sites with less adipose tissue. Functional experiments using patient‐derived tumour explants and macrophages isolated from malignant ascites further showed that blockade of the fatty‐acid transporter cluster of differentiation 36 or depletion of lipids from ascites reduced macrophage lipid loading and lipid oxidation‐associated stress, altered inflammatory cytokine production, and enhanced the expansion and effector activity of CD8^+^ T cells within omental metastatic tissues.^49^ These findings provide direct evidence that lipid uptake and processing can contribute to the functional remodelling of patient‐derived TAMs in OC.

The influence of hypoxia on TAM states is also supported by evidence from patients with HGSOC. A study performed single‐cell transcriptomic profiling of 34 samples collected from different anatomical sites in 17 patients with HGSOC and combined these data with spatial transcriptomics and multiplex IHC. As disease progressed from primary to metastatic sites, selenoprotein P^+^ macrophages with antigen‐presentation features progressively decreased, whereas *SPP1* ^+^ macrophages increased; the latter state was accompanied by stronger hypoxia, glycolysis and extracellular‐matrix degradation programmmes.^50^

Thus, TAM‐state heterogeneity in OC reflects not only transcriptional identity but also local lipid exposure, hypoxia and other lesion‐specific metabolic pressures.

### 3.6 Antitumour potential of natural killer cells

NK cells recognize abnormal cells by integrating signals from activating and inhibitory receptors and represent an important effector population in the innate antitumour immune response against OC. However, within malignant ascites and the local TME, NK cells are often functionally impaired. Patient‐derived studies have shown that NK cells in OC ascites can take up substantial amounts of local lipids, accompanied by alterations in lipid metabolism and effector programmes that impair their cytotoxic activity.^51^

Targeting iron metabolism in NK cells may potentially activate their function. Studies have shown that iron chelators can induce mitochondrial and DNA damage‐related stress in OC cells, enhance type I interferon responses, and exert NK cell‐dependent antitumour effects in a mouse model of metastatic OC.^52^ It should be noted that this mechanism is currently supported primarily by preclinical experiments; nevertheless, it suggests that iron metabolism can be regarded as a microenvironmental factor influencing the functional state of NK cells.

Cytokine support also plays an important role in shaping NK cell function. Interleukin‐15 (IL‐15) serves as a key regulator of NK cell survival, proliferation and cytotoxic activity. Studies using patient‐derived OC ascites have shown that IL‐15 stimulation enhances NK cell degranulation and IFN‐γ production and increases their cytotoxicity against OC cells.^53^

Overall, NK cell function is impaired in OC, yet there remains potential to restore their cytotoxic capacity.

## 4 BIOMARKER‐GUIDED PATIENT STRATIFICATION

The studies discussed above indicate that the OC immune microenvironment and the immune cells within it are neither static nor homogeneous, but rather continuously evolve across both spatial and temporal dimensions. This is also driving OC biomarker research to gradually shift from static, single‐dimensional detection toward multi‐layered and dynamic assessment, thereby informing patient stratification.

What now needs to be clarified is not merely ‘whether a tumour expresses a particular marker’, but rather what potential immunogenicity the tumour possesses, what immune state has formed, where these immune responses occur, and how they evolve with treatment.

### 4.1 Tumour‐intrinsic genomic determinants of immunogenicity

#### 4.1.1 Homologous recombination deficiency and breast cancer susceptibility genes alterations: Immunogenic potential

Homologous recombination deficiency (HRD) is a major molecular feature of HGSOC, most commonly arising from alterations in breast cancer susceptibility genes 1 and 2 (*BRCA1/2*) or other components of the homologous recombination repair pathway. Loss of *BRCA1/2* function compromises high‐fidelity repair of DNA double‐strand breaks and promotes the accumulation of structural variants and other genomic abnormalities.^54^ Such genomic instability provides a potential source of tumour neoantigens. It should be noted that these genomic alterations primarily represent the tumour's potential to develop immunogenicity. Whether this potential ultimately translates into an effective antitumour immune response relies on multiple factors, including antigen presentation, the functional state of immune cells, spatial organization and the local TME.

Studies using patient‐derived samples broadly support a link between HRD and increased immunogenicity. In HGSOC, Strickland et al. found that *BRCA1/2*‐mutated tumours had higher predicted neoantigen loads than tumours without identifiable alterations in homologous recombination genes. Higher neoantigen burden was associated with stronger expression of TCR, IFN‐γ, and tumour necrosis factor receptor pathway genes.^55^

HRD may increase tumour immunogenicity, but it does not guarantee the establishment of a sustained and effective antitumour immune response. In an integrative study of 160 tumour sites from 42 treatment‐naïve patients with HGSOC, *BRCA1*‐like and *BRCA2*‐like HRD tumours showed inflammatory signalling and evidence of ongoing immunoediting, but these features coexisted with loss of human leucocyte antigen diversity and infiltration by highly differentiated, dysfunctional CD8^+^ T cells.^56^ The immune state of an HRD‐positive lesion therefore needs to be assessed directly rather than inferred from genomic status alone.

Results from phase III clinical trials further highlight this limitation. DUO‐O reported PFS benefit in an HRD‐positive population within a multidrug regimen containing durvalumab, bevacizumab and olaparib.^57^ In contrast, ATHENA‐COMBO directly compared rucaparib plus nivolumab with rucaparib alone and did not demonstrate an additional PFS benefit from PD‐1 blockade, including in the HRD‐positive subgroup.^58^ These discordant findings do not negate the biological relationship between HRD and immunogenicity, but they argue against treating HRD as a stand‐alone, treatment‐specific biomarker.

A second mechanistic link between HRD and immune activation involves cytosolic DNA sensing through the cyclic GMP‐AMP synthase‐stimulator of interferon genes (STING)‐type I interferon pathway. Experimental studies have shown that unrepaired DNA damage can promote cytosolic DNA accumulation, STING‐dependent inflammatory signalling and immune‐cell recruitment.^59^ Nevertheless, direct causal evidence derived from human tumour lesions is still comparatively scarce.

Taken together, HRD is best regarded as a genomic marker reflecting the immunogenic potential of a tumour. It cannot replace direct evaluation of the actual immune status.

#### 4.1.2 Other genomic alterations and their immunological relevance

Beyond HRD, cyclin E1 (*CCNE1*) amplification, TP53 tumour‐suppressor gene (*TP53*) alterations and deficient mismatch repair/microsatellite instability‐high (dMMR/MSI‐H) represent genomic backgrounds with distinct immunological and clinical implications.

*CCNE1* amplification identifies a molecular subset of HGSOC that is frequently associated with relatively low immune infiltration. In a multicentre cohort of 348 patients with advanced HGSOC, tumours were classified according to *BRCA*, HRD and *CCNE1* status. *BRCA*‐altered tumours showed the highest immune‐cell densities, whereas *CCNE1*‐amplified tumours showed the lowest. Median OS was 52.5 months in the *BRCA*‐altered group compared with 28.0 months in the *CCNE1*‐amplified group.^60^ Thus, current patient‐level evidence supports an association between *CCNE1* amplification, relatively reduced immune infiltration, and adverse clinical outcome.

*TP53* is a different case. Integrated genomic analysis showed *TP53* mutations in approximately 96% of HGSOC, making *TP53* alteration a near‐universal molecular feature of this histological subtype.^61^ Consequently, a simple mutant‐versus‐wild‐type classification provides little discriminatory value for immune stratification within HGSOC. *TP53* is therefore better considered part of the underlying molecular context of HGSOC and interpreted together with HRD, *CCNE1* amplification, copy‐number patterns and the observed immune phenotype.

By contrast, dMMR/MSI‐H has more direct predictive relevance, although its importance in OC is strongly histotype dependent. In a group of 502 patients with early‐stage EOC in whom mismatch repair protein expression was assessed, mismatch repair deficiency was confined to endometriosis‐associated histologies, and no comparable enrichment was found in other major histological subtypes.^62^ In the phase II KEYNOTE‐158 study of previously treated advanced non‐colorectal MSI‐H/dMMR solid tumours, among 15 patients with MSI‐H/dMMR OC treated with pembrolizumab, 5 achieved an objective response, including 3 complete responses.^63^ Although this OC subgroup has a small sample size and the data originate from a pan‐cancer trial, dMMR/MSI‐H can nevertheless be regarded as a high‐value predictive biomarker in the small number of patients confirmed positive by testing.

### 4.2 Immune‐state and transcriptomic biomarkers

#### 4.2.1 Immune‐inflamed, immune‐excluded and immune‐desert phenotypes

The discussion above emphasizes that potential immunogenicity and the actual immune state established within a tumour are not the same concept. Immune cell states and transcriptomic biomarkers are complementary and can be used to more directly characterize the actual cellular composition, functional status, and spatial architecture of a given lesion at a specific time point. Within this framework, the commonly used categories of immune‐inflamed, immune‐desert, and immune‐excluded phenotypes can serve to summarize different tumour‐immune organizational patterns.

The core distinction among the immune‐inflamed, immune‐desert, and immune‐excluded phenotypes lies in the abundance of effector T cells, particularly CD8^+^ T cells, and their spatial localization relative to the tumour parenchyma and surrounding stroma.

The immune‐inflamed phenotype is marked by appreciable infiltration of CD8^+^ T cells into the tumour epithelium or tumour‐cell nests rather than their confinement to surrounding stromal areas. This spatial pattern is generally accompanied by evidence of active antigen presentation, interferon signalling, and cytotoxic T cell programmes. Adaptive inhibitory signals may also be increased in this context and can reflect negative feedback to an ongoing immune response.

In the immune‐excluded phenotype, immune cells are not absent from the TME; instead, they are largely confined to the tumour periphery or stromal compartments, with markedly limited penetration into the tumour epithelium or malignant cell nests. Thus, the defining feature is not a lack of immune recruitment, but spatial separation between effector lymphocytes and malignant cells. Stromal activation, extracellular matrix remodelling, cancer‐associated fibroblast (CAF)‐associated programmes, TGF‐β signalling, and abnormal vasculature may accompany this pattern.

The immune‐desert phenotype is characterized by absent or very low numbers of CD8^+^ T cells in the tumour parenchyma and surrounding stromal compartment. This phenotype is consistent with failure to generate or recruit a substantial effector T cell response and may be accompanied by weak antigen‐presentation, interferon, chemokine and cytotoxic transcriptional programmes.^64^

It should be particularly noted that no universally accepted numerical threshold currently exists to uniformly classify all patients into inflamed, excluded and desert phenotypes. Therefore, this tripartite classification is currently better suited as a relative spatial and functional framework. Importantly, immune‐inflamed, immune‐desert and immune‐excluded phenotypes should not be regarded as sharply separated biological states. An integrated digital pathology and transcriptomic analysis of 370 pretreatment OC specimens from the phase III ICON7 trial showed that both the overall abundance of CD8^+^ T cells and their distribution between tumour epithelium and stroma varied along a continuum rather than forming three sharply separated categories.^64^

This continuity and heterogeneity have prompted the development of more refined immune stratification approaches. Ghisoni et al. analysed 697 primary and recurrent OC specimens from 595 patients across five independent clinical cohorts. Based on the degree of CD8^+^ TIL infiltration and spatial heterogeneity, four immune phenotypes can be further identified: purely inflamed, mixed‐inflamed, excluded, and desert. This classification captured not only differences in T cell infiltration but also distinct spatial networks between TILs and myeloid cells. At the patient level, purely inflamed and mixed‐inflamed tumours were generally associated with more favourable survival, whereas excluded and desert phenotypes showed poorer outcomes.^65^

In general, the value of this classification resides in its ability to assess whether antitumour immunity has been initiated, whether effector cells can access and engage malignant cells, and where in the process the immune response principally breaks down.

#### 4.2.2 Predictive value and limitations of immune gene signatures and checkpoint expression

Transcriptomic immune signatures can further characterize whether a tumour harbours an ongoing T cell‐inflamed and adaptive immune response. Among these, the T‐cell‐inflamed gene expression signature (TIS) is one of the most extensively studied transcriptomic biomarkers. The classical 18‐gene TIS integrates multiple dimensions of antitumour immunity, including antigen presentation, T cell recruitment and activation, and adaptive immune suppression.^66^ Thus, The TIS reflects whether a relatively intact T cell–inflamed immune program has been established within the tumour.

In pan‐cancer KEYNOTE analyses, higher TIS scores correlated with an increased probability of response to pembrolizumab.^67^ By comparison, KEYNOTE‐100, which performed biomarker analyses in recurrent OC with evaluable RNA sequencing data from 317 patients, did not identify a clear association between TIS or other major gene expression signatures and pembrolizumab's ORR, PFS, or OS.^68^ Accordingly, in OC, TIS is currently better regarded as a research biomarker reflecting a pre‐existing T cell‐inflamed state rather than as a stand‐alone tool for selecting patients.

Immune checkpoint expression represents another important dimension for prediction. In KEYNOTE‐100, which enrolled 376 previously treated patients with recurrent OC, pembrolizumab monotherapy showed modest overall antitumour activity, while ORRs increased with higher PD‐L1 combined positive score (CPS), from approximately 5.0% in patients with CPS < 1 to 10.2% with CPS ≥ 1 and 17.1% with CPS ≥10.^7^

Patient‐derived studies further indicate that inhibitory immune states in OC are often mediated by multiple checkpoints rather than by a single pathway. Tumour‐derived NY‐ESO‐1‐specific CD8^+^ T cells have been shown to co‐express PD‐1 and LAG‐3 and to exhibit reduced IFN‐γ and tumour necrosis factor α production. In ex vivo experiments using patient‐derived T cells, simultaneous blockade of PD‐1 and LAG‐3 enhanced T cell proliferation and effector function.^69^ These findings support the involvement of multiple inhibitory receptors in T cell dysfunction.

Overall, TIS and immune‐checkpoint expression profiles provide information that is more closely related to the current functional immune state of the tumour. However, their predictive value in OC remains incompletely validated. These transcriptomic biomarkers cannot serve as independent determinants for immunotherapy decision‐making at present.

### 4.3 Multimodal and dynamic biomarkers: From spatiotemporal heterogeneity to longitudinal monitoring

Tissue‐based genomic, transcriptomic, and spatial immune biomarkers provide detailed information on the local TIME, but they share an important limitation: they are derived from restricted tissue sampling. This is particularly relevant in HGSOC, which is characterized by multifocal peritoneal dissemination and substantial interlesional heterogeneity. A single biopsy may therefore incompletely represent the overall disease state and cannot readily capture its evolution over time. Imaging and circulating biomarkers provide complementary information rather than substitutes for tissue analysis. Imaging extends assessment across multiple lesions and anatomical sites, whereas circulating biomarkers can track tumour burden, residual disease, and molecular evolution through serial sampling.

#### 4.3.1 Imaging and radiogenomics

Radiogenomics aims to establish links between macroscopic imaging features derived from computed tomography (CT) or magnetic resonance imaging and tumour genomic, transcriptomic and TIME characteristics. In OC, which is frequently multifocal and spatially heterogeneous, a single‐site tissue biopsy may not adequately represent the molecular and immune states of different lesions. Imaging, by contrast, can simultaneously assess the primary tumour and multiple metastatic sites, providing a potential non‐invasive approach for evaluating interlesional heterogeneity.

In patients with HGSOC, early studies have identified associations between CT imaging features, transcriptomic molecular subtypes, and clinical outcomes. For example, tumours with a high disease burden and extensive peritoneal or mesenteric dissemination have more frequently been associated with stromal‐related transcriptional features.^70^ These patient‐derived observations suggest that imaging phenotypes may indirectly reflect stromal composition and immune ecology.

With the development of radiomics, quantitative features such as texture, grayscale distribution, and interlesional heterogeneity have increasingly been used to predict tumour molecular states. Studies in patients with OC have developed contrast‐enhanced CT‐based models for predicting *BRCA* mutation status, while interlesional texture heterogeneity has also been associated with genomic features such as *CCNE1* amplification.^71^, ^72^

Associations between imaging features and immune infiltration have also received increasing attention. The CT‐based radiomic score developed by Sun et al. was associated with CD8^+^ T cell infiltration and clinical outcomes following anti‐PD‐1/PD‐L1 therapy.^73^ Although this study was primarily based on a multi‐cancer cohort, it suggests that similar models could be validated specifically in OC.

Artificial intelligence (AI) and convolutional neural networks may further facilitate automated imaging‐feature extraction and integration across multiple lesions. However, differences in imaging protocols, lesion segmentation, feature stability, cross‐centre reproducibility, and model interpretability remain major barriers to implementation.^74^

At present, radiomics and radiogenomics are better positioned as research tools for assessing whole‐body spatial heterogeneity and integrating multimodal biomarkers.

#### 4.3.2 Circulating biomarkers

Whereas imaging primarily expands the spatial coverage of biomarker assessment, the main advantage of circulating biomarkers lies in repeated sampling and temporal dynamic monitoring. Among these, circulating tumour DNA (ctDNA) has particularly strong potential for monitoring tumour burden, residual disease, recurrence and molecular evolution in OC.

Patient studies in HGSOC increasingly support this application. In a study of tumour‐derived ctDNA involving 27 patients with primary advanced HGSOC, the preoperative ctDNA detection rate reached 96%. On postoperative Day 10, ctDNA levels were significantly higher in patients with residual disease than in those who achieved macroscopic complete resection, suggesting that ctDNA may provide objective information on postoperative residual disease.^75^ In another study with serial blood sampling from 17 OC patients, ctDNA dynamics reflected treatment response; in some patients with sequential recurrence samples, ctDNA abnormalities appeared 2–5 months earlier than clinical, radiological or conventional biomarker changes.^76^

The CA‐125 elimination rate constant score (KELIM) also represents a dynamic biomarker. KELIM estimates intrinsic tumour chemosensitivity based on early CA‐125 decline kinetics during first‐line chemotherapy, and its prognostic value has been validated across multiple OC clinical trial datasets. Analyses of ICON7 and GOG‐0218 suggest that high‐risk patients with unfavourable KELIM may derive a relatively greater benefit from bevacizumab.^77^, ^78^ Therefore, KELIM is more appropriately positioned as a low‐cost, clinically accessible dynamic background indicator.

EVs, including commonly called exosomes, represent a potential source of liquid‐biopsy information in the malignant ascites and blood of patients with OC. EVs can carry tumour‐derived proteins, RNAs, and other molecular cargo. Patient‐derived studies have suggested that EVs contribute to local immune suppression. For example, ascites‐derived EVs have been indicated to suppress CD4^+^ and CD8^+^ T cell proliferation in vitro, suggesting a potential role in T cell dysfunction.^79^ However, evidence supporting their use for predicting immunotherapy response or monitoring immune states currently comes largely from pan‐cancer studies and exploratory analyses. In OC, EVs are better regarded as exploratory liquid‐biopsy biomarkers of tumour‐immune interactions and dynamic immune states, while their clinical value for response prediction, recurrence detection, or resistance monitoring still requires prospective validation.

### 4.4 From biomarker discovery to clinical implementation: A clinical‐use‐oriented framework

The genomic, transcriptomic, spatial, imaging and circulating biomarkers discussed above capture different dimensions of OC biology. Instead of treating various technologies as competing predictive modalities, a more rational approach is to stratify them based on clinical purpose, sample origin, level of validation, and barriers to implementation. Within this framework, different biomarkers may contribute to baseline characterization, response prediction, recurrence monitoring and assessment of acquired resistance (Table 3).

**TABLE 3:** Clinical‐use‐oriented framework for biomarkers in ovarian cancer.

Clinical use	Biomarker/modality	Core information	Validation status	Key limitation	References
Baseline characterization	*BRCA*/HRD	DNA‐repair deficiency	Clinically established molecular testing	Cannot measure actual immune state	^54^, ^55^, ^56^, ^57^, ^58^, ^59^
	*CCNE1* amplification	Low immune cell infiltration	Prognostic association; not an established immunotherapy predictor	Prospective interaction data lacking	^60^
	*TP53* alteration	Near‐universal HGSOC background	Diagnostic context	Little immune‐discriminatory value alone	^61^
	dMMR/MSI‐H	Identification of a small highly immunogenic subgroup	Established predictive value in confirmed positive tumours	Histotype‐dependent	^62^, ^63^
Baseline characterization/Response prediction	Spatial CD8^+^ phenotype	Local immune architecture and spatial accessibility	Supported by multiple patient cohorts	Does not standardized	^64^, ^65^
	TIS	Pre‐existing T‐cell‐inflamed state	Research marker	No clear ORR/PFS/OS association	^66^, ^67^, ^68^
	Checkpoint	Adaptive immune suppression	Driven by multiple checkpoints	Cannot generalize across regimens	^68^, ^69^
Multi Recurrence/Resistance	Serial ctDNA	Tracks residual disease, tumour burden, recurrence, and clonal evolution	Become abnormal earlier than expected	Absent intervention trials	^75^, ^76^
	KELIM	Estimate of early chemosensitivity	Validated prognostic/dynamic marker across ovarian trial datasets	Depends on standardized timing/modeling	^77^, ^78^
	EVs/exosomes	Potential tumour‐immune RNA/protein readout	Exploratory liquid biopsy biomarkers	Pending prospective validation	^79^
	Multi‐lesion imaging	Captures whole‐body burden and interlesional heterogeneity	Exploratory	Reproducibility and interpretability of the approach	^70^, ^71^, ^72^, ^73^, ^74^

#### 4.4.1 Baseline characterization: defining the molecular and immune context

Tumour tissue is the principal source for establishing the molecular and immune context of disease. *BRCA1/2* and HRD testing identifies DNA‐repair‐deficient tumours, but primarily reflects genomic background and potential immunogenicity rather than the immune state actually established within the tumour. DMMR/MSI‐H has more clearly established predictive relevance, although it occurs in only a small and histologically selected subset of OCs. These genomic markers should be used primarily to define the underlying biological context.

The distribution of CD8^+^ T cells between tumour epithelium and stroma, TIS and immune‐checkpoint expression can help determine whether a local antitumour immune response has actually developed. However, these biomarkers remain incompletely standardized with respect to sampling regions, analytical platforms and classification thresholds. In the near term, conventional molecular testing is better considered the baseline layer, with transcriptomic biomarkers serving as complementary tools for refining local immune phenotypes.

#### 4.4.2 Response prediction: Separating immunogenic potential from the realized immune state

Response prediction is one of the most active areas of biomarker research. At present, no single biomarker consistently predicts immunotherapy benefit in OC. HRD may indicate increased immunogenic potential, yet HRD‐positive tumours can still exhibit immune exclusion, impaired antigen presentation, or dysfunctional T cell states. PD‐L1 expression and TIS provide information on local immune activity, but their predictive performance in OC clinical studies has been inconsistent. Spatial CD8^+^ T cell profiling can distinguish immune infiltration from immune exclusion, but remains limited by single‐site tissue sampling.

A more appropriate approach is multilayered interpretation rather than the search for a single universal predictor. Biomarker assessment should distinguish whether a tumour has the molecular potential to generate an immune response, whether an active T cell response is actually present, and whether effector cells can physically access malignant‐cell compartments. Radiomics may further capture heterogeneity among different lesions.

#### 4.4.3 Recurrence monitoring: From static tissue biomarkers to longitudinal circulating markers

When the clinical question shifts to recurrence monitoring, the limitations of tissue‐based biomarkers become more pronounced, since repeated acquisition of tumour tissue is often impractical. In this setting, circulating biomarkers, which can be serially obtained, are more advantageous.

CtDNA can reflect tumour burden and clonal evolution and is emerging as a promising approach for minimal residual disease and molecular recurrence monitoring in OC. Serial ctDNA studies suggest that molecular abnormalities may precede radiographic or conventional serum‐marker evidence of recurrence in some patients.

KELIM also provides longitudinal information but reflects early CA‐125 clearance kinetics and intrinsic chemosensitivity rather than immune activity. EVs may capture additional tumour‐ and immune‐derived RNA or protein signals, but their use for recurrence monitoring remains at an exploratory stage. Unified standards for isolation and quantification are still absent.

#### 4.4.4 Resistance assessment: Identifying biological change

Resistance monitoring is fundamentally about identifying what changed and what did not after treatment. When tissue is available, re‐biopsy remains the most direct approach for evaluating newly acquired genomic alterations, antigen‐presentation defects, and spatial immune remodelling. Serial ctDNA can complement tissue analysis by tracking clonal evolution, whereas multi‐lesion imaging can reveal spatially discordant patterns of progression.

An optimal framework for resistance would integrate longitudinal tissue, circulating and imaging information. In this setting, AI and multi‐omic approaches are most useful as integration tools that combine data across different lesions, sample types, and time points into patient‐level dynamic models.

Future research should continue to focus on prospective, multicentre validation of biomarkers. The clinical translational value of a biomarker can only be truly established when it provides incremental information beyond existing standards and actually changes patient management.

## 5 EMERGING THERAPEUTIC PLATFORMS AND COMBINATION STRATEGIES GUIDED BY SPATIOTEMPORAL HETEROGENEITY

**FIGURE 4:**
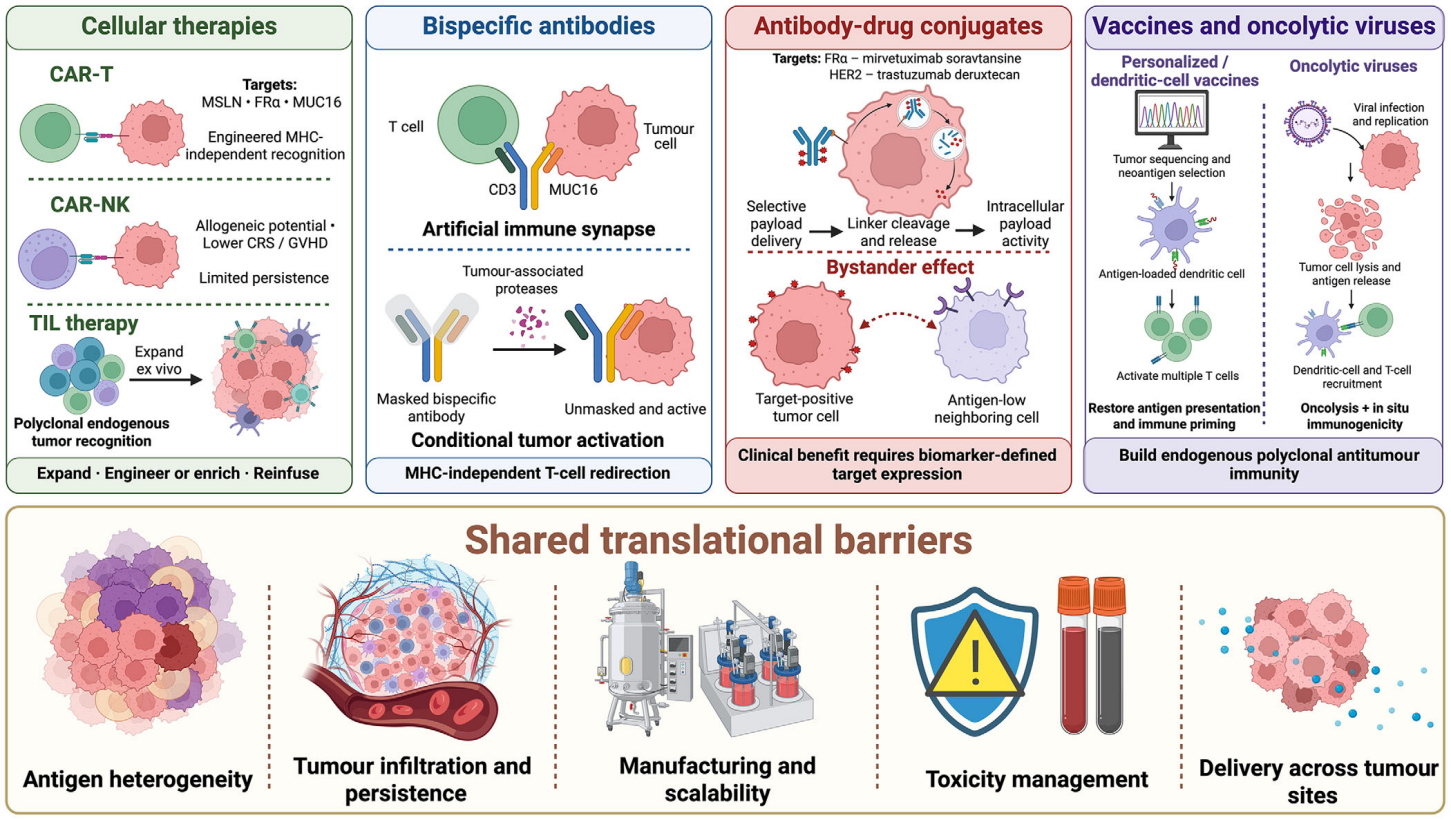
Emerging immunotherapeutic platforms for ovarian cancer. Cellular therapies, bispecific antibodies, antibody–drug conjugates, tumour vaccines, and oncolytic viruses provide complementary strategies to enhance immune activation and tumour‐selective cytotoxicity.

The persistent spatial differences and temporal evolution not only explain the immune heterogeneity of OC but also place higher demands on the development of novel therapies and the design of combination strategies (Figure 4, Table 4). Treatment approaches are gradually shifting from traditional empirical drug selection toward differentiated interventions tailored to specific tumour states.

**TABLE 4:** Clinical trial designs, treatment regimens and outcomes of immunotherapy‐based strategies in ovarian cancer.

Identifier	Design/population	Regimen/comparator	Core endpoint	Key efficacy data	Biomarker stratification	Conclusion/status	References
NCT02718417	Phase III; *n* = 998; newly diagnosed stage III‐IV EOC	Chemotherapy +/‐ concurrent and/or maintenance avelumab	Primary: BICR PFS	PFS: 16.8 mo. (maintenance), 18.1 mo. (concurrent + maintenance), NE (control); HR 1.43 and 1.14 vs. control. OS not definitively assessed.	Unselected; PD‐L1 exploratory	Negative; stopped for futility	^8^
NCT03038100	Phase III; *n* = 1301; newly diagnosed stage III–IV OC	Atezolizumab + carboplatin/paclitaxel + bevacizumab, then atezolizumab + bevacizumab vs placebo regimen	Coprimary: PFS and OS in ITT and PD‐L1^+^ populations	PFS: 19.5 vs. 18.4 mo. (HR 0.92); OS: 50.5 vs. 46.6 mo. (HR 0.92). PD‐L1^+^ PFS HR 0.80; OS HR 0.83.	PD‐L1 SP142 IC ≥1% prespecified; IC ≥5% signal exploratory	Negative; no significant PFS or OS benefit.	^80^
NCT03737643	Phase III; *n *= 1130; newly diagnosed non‐tBRCAm advanced OC	Chemotherapy + bevacizumab + durvalumab, then durvalumab + bevacizumab + olaparib vs control regimen	Primary: PFS in HRD^+^ and ITT populations	PFS: 45.1 vs. 23.3 mo. in HRD^+^ (HR 0.46); 25.1 vs. 19.3 mo. in ITT (HR 0.61). Interim OS HR 0.95.	Non‐tBRCAm; HRD prespecified	PFS positive; durvalumab effect cannot be isolated without an olaparib‐only arm	^57^
NCT02891824	Phase III; *n* = 614; platinum‐sensitive recurrent nonmucinous EOC	Atezolizumab + platinum doublet + bevacizumab, then atezolizumab + bevacizumab vs placebo regimen	Coprimary: PFS in ITT and PD‐L1^+^ populations	PFS: 13.5 vs 11.3 mo. (HR 0.83; prespecified significance not met); immature OS: 35.5 vs. 30.6 mo. (HR 0.81).	PD‐L1 IC ≥1% prespecified; not predictive	PFS negative; numerical OS trend remains nonconfirmatory	^81^
NCT03598270	Phase III; *n* = 417; late‐relapsing platinum‐sensitive recurrent high‐grade OC	Atezolizumab + carboplatin doublet, then atezolizumab + niraparib vs placebo regimen	Primary: investigator‐assessed PFS	ORR: 45% vs. 43%; PFS: 11.2 vs. 10.1 mo. (HR 0.89)	*BRCA* and PD‐L1 IC < 1%/≥1% prespecified; no predictive subgroup	Negative; no significant PFS or ORR improvement	^82^
NCT02580058	Phase III; *n *= 566; platinum‐resistant/refractory disease	Avelumab + PLD vs. avelumab vs. PLD	Co‐primary: BICR PFS and OS	ORR: 13.3% / 3.7% / 4.2%; PFS: 3.7 / 1.9 / 3.5 mo.; OS: 15.7 / 11.8 / 13.1 mo.	PD‐L1 exploratory; no validated cut‐off	Negative for PFS and OS	^9^
NCT05116189	Phase III; *n* = 643; platinum‐resistant recurrence after 1–2 lines	Pembrolizumab + weekly paclitaxel +/‐ bevacizumab vs placebo regimen	Primary: PFS; key secondary: OS	CPS≥1: ORR 53.0% vs. 46.6%; PFS 8.3 vs 7.2 mo. (HR 0.72); OS 18.2 vs. 14.0 mo. (HR 0.76). ITT PFS HR 0.70; OS HR 0.82	CPS≥1; FDA‐authorized 22C3 assay.	Positive; FDA‐approved in 2026 for the CPS≥1 population	^83^
NCT02498600	Randomized phase II; *n *= 100; recurrent/persistent EOC	Nivolumab + ipilimumab induction vs. nivolumab	Co‐primary: ORR and PFS	ORR: 31.4% vs. 12.2%; PFS: 3.9 vs 2.0 mo. (HR 0.53); OS: 28.1 vs. 21.8 mo. (HR 0.79; NS)	PD‐L1 exploratory; not predictive	Improved ORR/PFS; limited durability and no significant OS benefit	^84^
NCT04739800	Randomized phase II; *n* = 153; platinum‐resistant recurrence after bevacizumab	SOC chemotherapy vs durvalumab + olaparib + cediranib vs. durvalumab + cediranib vs. olaparib + cediranib	Primary: PFS	PFS: 3.4 / 2.9 / 2.5 / 2.8 mo.; OS: 7.5 / 8.3 / 5.7 / 10.2 mo. (SOC / triplet / durvalumab doublet / olaparib doublet)	Prior PARPi/ICI exposure stratified; no validated predictive marker	Negative; all experimental arms failed superiority and the trial stopped for futility	^85^
NCT03574779	Single‐arm phase II; *n *= 41; pretreated platinum‐resistant OC	Niraparib + dostarlimab + bevacizumab	Primary: ORR and safety	ORR 17.1%; DCR 73.2%; PFS 7.9 mo.; OS 22.1 mo.	*BRCA*/HRR/HRD/PD‐L1 exploratory; none correlated with response	Modest activity; no validated selection marker	^86^
NCT02606305	Phase Ib expansion; *n *= 56; heavily pretreated FRα‐expressing platinum‐resistant OC	Mirvetuximab soravtansine + pembrolizumab	Primary: safety/tolerability and ORR	ORR 31%; DOR 8.0 mo.; PFS 4.2 mo.	FRα expression required/evaluated; no validated ICI‐specific marker	Active and tolerable, but added immune synergy remains unproven	^87^
NCT02107950	Randomized phase II; *n *= 71; first platinum‐sensitive relapse	DCVAC/OvCa + carboplatin/gemcitabine vs chemotherapy	Primary: PFS	ORR: 87.5% vs. 62.5%; PFS: 11.3 vs 10.1 mo. (HR 0.73; NS); OS: 35.5 vs. 22.1 mo. (exploratory)	No validated marker; immune correlates exploratory	PFS negative; OS signal requires confirmation	^88^
NCT02759588	Single‐arm phase II; *n* = 27; heavily pretreated platinum‐resistant/refractory OC	Intraperitoneal Olvi‐Vec followed by platinum doublet +/‐ bevacizumab	Primary: ORR and PFS	ORR 54%; DCR 88%; DOR 7.6 mo.; PFS 11.0 mo.; OS 15.7 mo.	No validated predictive marker	Hypothesis‐generating; confirmatory randomized evidence required	^89^

*Note*: The above data were obtained from the ClinicalTrials.gov website.

Abbreviations: BICR, blinded independent central review; tBRCAm, tumour *BRCA* mutation; CPS, combined positive score; DCR, disease control rate; DOR, duration of response; HR, hazard ratio; HRR, homologous recombination repair; SP142, PD‐L1 immunohistochemistry antibody clone; IC, immune‐cell score for PD‐L1 expression; ITT, intention‐to‐treat; LOH, loss of heterozygosity; NE, not estimable; NS, not significant; ORR, objective response rate; PLD, pegylated liposomal doxorubicin; SOC, standard of care; 22C3, PD‐L1 immunohistochemistry antibody clone; DCVAC/OvCa, autologous dendritic‐cell vaccine product for ovarian cancer.

### 5.1 Emerging therapeutic platforms

#### 5.1.1 Cellular therapies

Adoptive cell therapy, which includes the ex vivo expansion, activation, or genetic modification of immune cells followed by reinfusion into patients, provides an approach to overcome insufficient endogenous immune priming and impaired effector‐cell function in OC. The major approaches currently include chimeric antigen receptor T (CAR‐T) cells, chimeric antigen receptor natural killer (CAR‐NK) cells, and TIL therapy. These modalities exploit engineered antigen recognition, the intrinsic cytotoxicity of NK cells, and endogenous polyclonal tumour‐reactive lymphocytes, respectively and offer distinct advantages in addressing defective antigen presentation and tumour heterogeneity.

CAR‐T cells directly recognize tumour‐surface antigens through engineered chimeric antigen receptors and do not require conventional major histocompatibility complex (MHC)‐dependent antigen presentation, providing a potential advantage in tumours with impaired antigen‐presentation capacity. Current OC studies have predominantly focused on mesothelin (MSLN), folate receptor α (FRα), and mucin 16 (MUC16). MSLN‐directed CAR‐T cells have entered early clinical investigation, although heterogeneous antigen expression and treatment‐induced antigen loss may facilitate tumour escape^.^ ^90^ FRα and MUC16 also represent attractive tumour‐associated targets, but CAR‐T approaches directed against these targets remain in need of further clinical validation.^91^, ^92^ Moreover, in solid tumours, dense ECM, abnormal vasculature, and persistent immunosuppressive signalling restrict CAR‐T cell infiltration and persistence. Preclinical studies have shown that CAR‐T cells overexpressing transgelin 2 and engineered CAR‐T cells secreting interleukin‐7/C‐C motif chemokine ligand 19 can improve tissue infiltration and local immune recruitment, representing important directions for enhancing CAR‐T efficacy in the future.^93^

CAR‐NK cells provide an alternative approach by exploiting the MHC‐independent cytotoxicity of NK cells. Their relatively low risks of cytokine release syndrome (CRS) and graft‐versus‐host disease, together with their compatibility with allogeneic manufacture, make them particularly attractive for the development of cellular products.^94^ Cord blood‐ and induced pluripotent stem cell‐derived CAR‐NK cells may offer additional advantages in batch standardization and scalable manufacturing,^95^ and MSLN‐directed induced pluripotent stem cell‐CAR‐NK cells have shown antitumour activity with favourable safety profiles in preclinical OC models.^96^ Nevertheless, limited in vivo persistence remains a major limitation. IL‐15 or IL‐15/IL‐15 receptor α co‐expression strategies are being explored to improve expansion and long‐term functionality.^97^

TIL therapy differs from CAR‐based approaches by exploiting naturally occurring polyclonal tumour‐reactive lymphocytes derived directly from the tumour. This polyclonality enables recognition of multiple endogenous antigens and may reduce the likelihood of therapeutic escape caused by loss of a single target. Preliminary studies have reported disease control in patients with advanced or platinum‐resistant OC following expansion of tumour‐reactive TILs.^98^ However, truly tumour‐reactive lymphocytes constitute only a fraction of the total TIL population, and their identification, isolation and selective expansion remain technically demanding.^99^ Current optimization strategies therefore focus on refining lymphodepletion, enriching highly reactive T cell clonotypes, and combining TIL therapy with other interventions that preserve the function of transferred lymphocytes

Despite their biological promise, cellular therapies face substantial translational barriers related to manufacturing complexity and cost. Autologous CAR‐T and TIL products require multiple steps, including tissue or cell procurement, ex vivo expansion, genetic modification where applicable, quality‐control testing, and reinfusion.^100^, ^101^ Long manufacturing times and inter‐batch variability can be particularly problematic in patients with rapidly progressive platinum‐resistant disease.^102^ In addition, CRS, neurotoxicity, complications associated with lymphodepletion, and cytokine‐support regimens require specialized inpatient monitoring and toxicity‐management infrastructure.^103^ Consequently, successful translation of cellular therapy into routine OC care will depend not only on antitumour efficacy but also on the development of reproducible, scalable, and economically sustainable manufacturing and delivery systems.

#### 5.1.2 Bispecific antibodies and antibody‐drug conjugates

Advances in antibody engineering have further expanded precision therapeutic options for OC. Bispecific antibodies redirect immune effector cells toward tumour cells through simultaneous recognition of tumour‐associated and immune‐cell antigens, whereas antibody‐drug conjugates (ADCs) exploit tumour‐surface antigens to selectively deliver highly potent cytotoxic payloads.

Bispecific T‐cell engagers represent one of the most extensively developed classes of bispecific antibodies. By simultaneously engaging a tumour‐associated antigen and cluster of differentiation 3, bispecific T‐cell engagers establish an artificial immune synapse and trigger T cell‐mediated cytotoxicity independently of conventional MHC‐mediated antigen presentation.^104^ MUC16/cluster of differentiation 3‐directed agents such as REGN4018 have demonstrated the ability to activate T cells and induce tumour‐cell killing, although clinical development in OC remains at an early stage.^105^ Major limitations include low‐level target expression in normal tissues, systemic T cell activation and CRS. Early epithelial cell adhesion molecule‐targeting approaches, for example, were limited by narrow therapeutic windows. To improve safety, newer bispecific platforms increasingly incorporate conditional activation mechanisms, including protease‐cleavable masking domains that preferentially activate the molecule within the TME.^106^ Such designs reflect a broader transition from simply maximizing T cell activation toward improving tumour selectivity and therapeutic index.

In contrast, ADCs have achieved more definitive clinical validation in OC. FRα‐directed mirvetuximab soravtansine represents the most prominent example. In the phase III MIRASOL trial, mirvetuximab soravtansine promoted progression‐free and OS compared with chemotherapy in patients with FRα‐positive platinum‐resistant OC.^107^ Importantly, this clinical benefit is predicated on biomarker‐driven patient selection. However, FRα testing methods and scoring criteria are not fully harmonized, patient selection remains a clinical challenge. Other ADCs, like trastuzumab deruxtecan, can further kill adjacent antigen‐negative tumour cells through its cleavable linker.^108^ ADC clinical translation also has clear limitations. Their manufacture requires precise control of antibody specificity, linker stability, drug‐to‐antibody ratio and cytotoxic payload characteristics, each of which may influence efficacy and toxicity.^109^ Clinical activity also depends heavily on target‐antigen expression, necessitating reliable companion diagnostics and reproducible definitions of biomarker positivity. Spatial heterogeneity of target expression and treatment‐associated antigen downregulation may contribute to acquired resistance.

Neither bispecific antibodies nor ADCs can completely escape the influence of the local immune ecosystem. Profound T‐cell exhaustion, myeloid suppression, and stromal exclusion may all attenuate therapeutic efficacy. In the future, these modalities will likely need to be combined with other treatments to improve outcomes.

#### 5.1.3 Tumour vaccines and oncolytic viruses

In contrast to therapies that directly supply effector cells or cytotoxic payloads, tumour vaccines and oncolytic viruses (OVs) primarily aim to restore endogenous antitumour immunity by improving antigen presentation and local inflammatory signalling. These strategies may therefore be particularly relevant to ovarian tumours characterized by deficient immune priming and limited T‐cell infiltration.

By targeting patient‐specific mutations, neoantigen‐based personalized vaccines elicit highly individualized T cell responses, theoretically minimizing off‐target toxicity while enhancing the quality of antitumour immunity. Advances in high‐throughput sequencing and mRNA delivery have improved the feasibility of individualized vaccine design and enabled the induction of polyclonal T cell responses with memory‐like features.^110^ DC‐based vaccines similarly promote endogenous T cell activation by enhancing tumour‐antigen presentation. In a phase II study, the representative autologous DC vaccine product for OC, when combined with chemotherapy did not significantly improve PFS in patients with platinum‐sensitive OC. However, exploratory analyses suggest a potential OS benefit and enhanced antigen‐specific immune responses.^88^ Currently, the main limitation of tumour vaccines in OC lies in their clinical translational efficiency. Personalized vaccines require tumour sequencing, human leukocyte antigen typing, neoantigen prediction, and customized manufacturing; the production timeline, cost, and the actual immunogenicity of predicted antigens may all affect their accessibility and efficacy.^110^

OVs can selectively lyse tumour cells while promoting local antigen presentation and inflammatory responses through the induction of immunogenic cell death and the release of tumour antigens and damage‐associated molecular patterns. In a phase II trial of platinum‐resistant OC, Olvi‐Vec exhibited acceptable safety and preliminary antitumour activity, with evidence of prolonged PFS and notable immunostimulatory potential, even though its OS benefit did not attain statistical significance.^89^ More recently, OV development has shifted from direct oncolysis alone toward immune modulation. For example, MEM‐288 can express immunostimulatory molecules such as interferon β and cluster of differentiation 40 ligand to enhance T cell recruitment and activation.^111^ However, these approaches still face important translational challenges, including scalable viral manufacturing, host antiviral immunity, biosafety, viral shedding and heterogeneous delivery across tumour sites.

### 5.2 Lessons from phase III trials: From empirical combinations to context‐dependent immunotherapy

For OC, the translation of immunotherapy in large randomized clinical trials has not been smooth. Several early phase III studies attempted to incorporate immune checkpoint inhibitors (ICIs) directly into established treatment frameworks but overall failed to achieve the anticipated benefit. JAVELIN Ovarian 100 evaluated avelumab either as maintenance following chemotherapy or in combination with first‐line platinum‐based chemotherapy in patients with newly diagnosed advanced EOC. Neither strategy improved PFS, and the trial was discontinued for futility.^8^ In platinum‐resistant or platinum‐refractory disease, JAVELIN Ovarian 200 further compared avelumab monotherapy, avelumab plus PLD, and PLD alone, but similarly failed to meet its primary PFS and OS endpoints.^9^

These findings suggest that the limited success of immunotherapy in OC cannot be attributed simply to insufficient activity of checkpoint blockade itself but may instead reflect a mismatch between therapeutic mechanism and the tumour ecological state present at the time of treatment.

Previous phase III trials generally enrolled broad patient populations with highly heterogeneous molecular and immune backgrounds. They exhibit different sensitivities to immunotherapy. In largely unselected populations, relatively small immune‐responsive subgroups may have been diluted by a larger proportion of tumours with limited intrinsic susceptibility to checkpoint blockade.

Treatment timing may also be as important as the therapy itself. The immune microenvironment differs substantially among treatment‐naïve tumours, residual disease after chemotherapy, and platinum‐resistant recurrent lesions. A fixed ‘chemotherapy plus ICI’ strategy applied across the disease course may fail to exploit transient windows of immune susceptibility. Spatial heterogeneity further complicates this issue. Primary tumours, peritoneal and omental metastases, and malignant ascites can differ in T cell distribution, stromal barriers and myeloid composition. A single biopsy or a single PD‐L1 test can hardly represent the immune ecosystem of all lesions in a patient.

More importantly, mechanistic synergy does not necessarily imply mechanistic dependency within an individual tumour. Chemotherapy can increase antigen release, antiangiogenic therapy can improve abnormal vasculature, and PARPis can promote DNA‐damage‐associated immune activation. However, if the dominant barrier within a particular lesion is CAF/ECM‐mediated immune exclusion, myeloid‐dominant suppression, or terminal T cell exhaustion, simply combining PD‐1/PD‐L1 blockade with the agents discussed above may still be insufficient to restore complete antitumour immunity.

Therefore, the design of combination therapy should not be based solely on whether two agents are theoretically synergistic at the population level; rather, it should first identify the dominant immune escape mechanisms operating in the current disease stage and in specific lesions. This concept is beginning to gain support from more recent clinical evidence. In contrast to earlier ICI trials conducted in broadly unselected populations, KEYNOTE‐B96 evaluated pembrolizumab combined with weekly paclitaxel, with or without bevacizumab, in a clearly defined platinum‐resistant setting and demonstrated improvements in PFS and OS among patients with PD‐L1 CPS ≥1,^83^ ultimately leading to United States Food and Drug Administration approval of the regimen for this biomarker‐defined population in 2026.^112^

Accordingly, future treatment design should distinguish the dominant rate‐limiting processes across different tumour states and incorporate biomarker‐based stratification, microenvironmental features, and temporal disease evolution into both combination therapy and treatment sequencing.

### 5.3 Precision combination therapy based on patient stratification and spatiotemporal heterogeneity

Precision combination therapy needs to sequentially address these questions: defining the biological complementarity between different treatments; selecting differential intervention strategies based on immune ‘hot’ versus ‘cold’ states; dynamically adjusting treatment sequencing while considering spatial and temporal heterogeneity and re‐identifying the dominant therapeutic barriers after resistance develops.

#### 5.3.1 Combination therapy based on mechanistic complementarity

Mechanistic complementarity does not simply mean stacking multiple agents with antitumour activity; rather, it refers to establishing synergy by targeting distinct limiting steps within the cancer‐immunity cycle.

The combination of ICIs is primarily based on their complementary functions at different stages of immune suppression. PD‐1 mainly restrains the effector function of activated T cells within the TME. CTLA‐4 acts predominantly during early T cell activation and clonal expansion. Dual blockade can relieve inhibitory signals at distinct stages of the T cell response. In a randomized phase II study, nivolumab plus ipilimumab achieved a higher response rate and longer PFS than nivolumab alone in recurrent or persistent OC.^84^ Other checkpoints, including LAG‐3, T‐cell immunoglobulin and mucin‐domain containing 3, and TIGIT, may contribute to persistent or compensatory immune suppression. On dysfunctional T cells or NK cells, they are frequently co‐expressed with PD‐1. Preclinical studies suggest that blockade of these pathways can restore effector lymphocyte function.^113^

The synergy between targeted therapy and immunotherapy is also an important direction. PARP inhibition can activate cyclic GMP‐AMP synthase‐STING‐related signalling through DNA damage and cytosolic DNA accumulation. This signalling promotes type I interferon production and CD8^+^ T cell recruitment. ICIs can then relieve PD‐1/PD‐L1‐mediated T cell suppression, allowing PARPi‐induced immune priming to be converted into sustained effector T cell cytotoxicity.^114^, ^115^ For antiangiogenic therapy, it can improve T cell recruitment and infiltration into tumour tissue. Motz et al. found that the tumour vascular endothelium in OC highly expresses Fas ligand, which selectively restricts CD8^+^ T cell entry into tumours. The VEGF is involved in maintaining this immune barrier. Inhibition of the VEGF/Fas ligand axis can increase intratumoral CD8^+^ T cell infiltration.^116^ On this basis, ICIs can subsequently relieve checkpoint‐mediated inhibition of these infiltrating T cells, restoring their cytotoxic activity and cytokine production. This strategy has a strong combinatorial rationale.^117^

Chemotherapy can induce a transient state of immune activation in some patients, providing a more favourable basis for ICI action. However, this effect is not sustained. As treatment progresses, immunosuppressive mechanisms such as T cell exhaustion may re‐emerge and intensify. Therefore, the synergy between chemotherapy and ICIs is markedly time dependent. Dose‐dense chemotherapy, which employs a weekly low‐dose schedule, can not only reduce severe adverse effects such as myelosuppression and alopecia, but also help maintain the overall stability of immune system function.^118^ It represents not merely a pharmacokinetic optimization, but also a potentially ‘immune‐friendly’ form of chemotherapy, providing a favourable foundation for combination with immunotherapy. Hyperthermic intraperitoneal chemotherapy (HIPEC) is a locoregional treatment modality. Through local hyperthermia and high‐concentration chemotherapy, HIPEC can enhance tumour cell damage and further induce heat stress and inflammatory responses, thereby altering the peritoneal TME. Preclinical studies have suggested that HIPEC synergizes with PD‐1 blockade to produce antitumour effects in the mouse model of colorectal cancer peritoneal metastasis.^119^ Considering that OC disseminates primarily within the peritoneal cavity, the combination of HIPEC and ICIs holds considerable therapeutic potential.

#### 5.3.2 Differential intervention for immune‐hot and immune‐cold tumours

The therapeutic goals for immune‐hot and immune‐cold tumours are distinct: in the former, the emphasis is on amplifying existing immune responses. In the latter, the aim is to establish an effective immune response or to remove microenvironmental barriers that prevent effector cell function.

For immune‐hot tumours, reversing T cell exhaustion is central to treatment. Single‐agent ICB is often insufficient to revive T cell function, and multiple checkpoint blockade is the most considered strategy. On this basis, effector function can be further enhanced according to accompanying molecular features. For example, PARPi‐based regimens in HRD‐positive patients, or antiangiogenic therapy in patients with prominent angiogenic features.

For immune‐cold tumours, the therapy focuses on converting them into a responsive state. In tumours with a marked paucity of T cells, strategies such as cancer vaccines, OVs, and chemotherapy may be considered to promote immune priming and T cell recruitment. In tumours where T cells are present but confined to the tumour periphery or stromal regions, antiangiogenic therapy and microenvironment‐targeted interventions against TGF‐β, CAFs, or the ECM may be prioritized to enhance the ability of immune cells to penetrate the tumour parenchyma.

#### 5.3.3 Spatiotemporal immune heterogeneity as a basis for dynamic treatment adaptation

The strategies for immune‐hot and immune‐cold tumours provide a basic therapeutic framework. However, this framework rests on an important premise: the assessed immune state is representative of the patient's overall disease. In fact, the immune ecosystem of OC exhibits marked spatiotemporal heterogeneity. The treatment selection based on immune status must incorporate two additional dimensions: sampling site and treatment timing.

Spatial heterogeneity may modify treatment priorities within the same patient. A primary tumour with abundant intratumoral T cells may provide a stronger rationale for checkpoint‐based therapy. When omental or peritoneal metastases are dominated by immune exclusion, selecting ICI therapy based solely on the primary lesion may overestimate overall treatment sensitivity. In this setting, combination strategies incorporating microenvironment remodelling should be prioritized. Malignant ascites offers additional value by supplementing tissue evaluation and providing a dynamic readout of peritoneal T cell status and its changes over time. For recurrent lesions, the immune state may already differ from the primary tumour. Treatment decisions cannot rely solely on archival tissue obtained at initial diagnosis. Greater consideration should be given to the actual status of the current lesion. It should be emphasized that these differences are not sufficient to establish a fixed ‘site‐drug’ correspondence. A more reasonable principle is to prioritize treatment according to the dominant immune barrier within the current disease burden.

Temporal heterogeneity determines when treatment should be initiated and when it should be adjusted. Strategies based on the immune status of the original lesion should not be maintained throughout the entire treatment course. Some tumours with weak immune responses before treatment may exhibit more pronounced inflammatory activation and T cell infiltration after chemotherapy, potentially creating a therapeutic window that is more suitable for ICI intervention. If immune exclusion remains dominant after chemotherapy, simply intensifying checkpoint blockade may yield limited benefit. In this setting, priority should be given to addressing the major microenvironmental barriers that persist. Similarly, once the disease enters the recurrent stage, treatment regimens should be reassessed according to the current disease state.

#### 5.3.4 Re‐stratification and treatment adaptation after resistance

Drug resistance remains a central challenge limiting long‐term outcomes in OC. It encompasses primary and acquired resistance. Primary resistance means the failure to achieve an effective response from the outset of treatment, whereas acquired resistance refers to the gradual loss of treatment response after initial sensitivity.

One of the bases of primary resistance is the pre‐existence of resistant clones before treatment. Genomic studies suggest that some treatment failures do not result exclusively from newly acquired mutations but rather from the selection of low‐frequency resistant clones already present at diagnosis. *CCNE1* amplification, for example, is enriched in primary platinum‐resistant or refractory HGSOC.^120^ Accordingly, identification of this resistance risk should be moved as early as possible before treatment initiation. Primary resistance may also be supported by a pre‐existing protective TME. In OC models and patient‐derived materials, CAFs can reduce the effective accumulation of platinum drugs in tumour cells by altering glutathione and cysteine metabolism, contributing to platinum resistance.^121^ In terms of treatment, the priority in primary resistance is to redirect therapy early, before sustained ineffective treatment leads to the selection of resistant clones.

Acquired resistance develops after an initially effective response and is driven by genetic and functional adaptation of residual tumour cells under sustained therapeutic pressure. In HGSOC, acquired resistance mechanisms supported by evidence from patient samples include *BRCA1/2* reversion mutations, loss of BRCA1 or RAD51 paralog C promoter methylation, and ATP‐binding cassette subfamily B member 1‐associated structural rearrangements. The former two mechanisms can restore homologous recombination repair and reduce sensitivity to platinum agents and PARPis. ATP‐binding cassette subfamily B member 1 rearrangements can promote chemotherapy resistance by increasing multidrug resistance protein 1 expression and drug efflux.^120^ Beyond acquired genetic alterations, therapeutic pressure may also promote resistance through non‐genetic adaptation. In OC models, prolonged drug exposure has been associated with epithelial‐mesenchymal transition‐like transitions, bypass pathway activation, and epigenetic reprogramming.^122^ Dynamic monitoring is central to the management of acquired resistance. In *BRCA1/2*‐mutated high‐grade OC, circulating cell‐free DNA (cfDNA) analysis can be used to detect *BRCA* reversion mutations associated with resistance to platinum agents and PARPis. Lin et al. reported that *BRCA* reversion mutations were detectable in cfDNA before rucaparib treatment in a subset of patients with platinum‐resistant or platinum‐refractory disease.^123^ These findings support the potential utility of cfDNA for longitudinally tracking dynamic changes in *BRCA*‐associated resistant clones.

Overall, the management of drug resistance in OC should establish a continuous management model comprising ‘resistance risk identification‐dynamic monitoring‐mechanism confirmation‐treatment adjustment’, providing more durable long‐term treatment strategies for patients with OC.

## 6 CHALLENGES AND FUTURE DIRECTIONS FOR CLINICAL TRANSLATION

As the dimensionality of available information increases, the ability to translate complex research findings into robust, interpretable and reproducible clinical tools has become a major barrier to further progress. Future translational efforts should shift from continuously expanding the number of candidate markers toward establishing an integrated pathway encompassing assay standardization, multimodal data integration, external validation and assessment of clinical decision utility.

### 6.1 Biomarker standardization

Clinical translation of a biomarker requires more than a statistically significant association. The measurement must remain stable and interpretable across time points, platforms and institutions. OC biomarker studies continue to show substantial heterogeneity in sample collection, storage, preprocessing, analytical platforms and threshold definition. Blood‐based markers such as lysophosphatidic acid, for example, can be affected by pre‐analytical factors including storage duration and anticoagulation conditions.^124^ Biomarker standardization should encompass the entire analytical pathway, from specimen acquisition and processing to assay performance and result interpretation.

External validation is equally important for distinguishing exploratory biomarkers from clinically actionable tools. Models developed in single‐centre may capture cohort‐specific characteristics, and their apparent diagnostic or predictive performance may not be fully reproduced in independent populations. Multicentre validation not only establishes the robustness of candidate biomarkers across different populations but also provides an important foundation for subsequent assay standardization and clinical implementation. For example, a 2025 multicentre study involving 1183 women with adnexal masses from four hospitals showed that the serum small EV‐based Ovarian Cancer Score, incorporating CA‐125, human epididymis protein 4, and complement component 5a, maintained high diagnostic performance across centres, supporting the value of multicentre validation for assessing biomarker robustness and translational potential.^125^

Overall, rigorous standardization and multicentre validation are essential for transforming promising OC biomarkers from exploratory findings into reproducible and clinically implementable tools.

### 6.2 AI‐assisted precision immunotherapy

The potential value of AI lies not simply in replacing conventional statistical analyses, but in transforming multidimensional data into predictions with clear clinical relevance.

An implementable AI framework should first clearly define its inputs and outputs. In OC, model inputs may include clinical variables such as age, stage, and prior treatment; haematoxylin and eosin (H&E) or multiplex IF images; molecular features such as *BRCA*/HRD status; and transcriptomic or immune‐related expression features. At the research stage, single‐cell and spatial‐transcriptomic data may also be incorporated to provide more refined immune‐state labels. Single‐cell and spatial studies in OC have already characterized features like T cell exhaustion, TAM enrichment and spatial immune exclusion, providing a biological basis for establishing such reference phenotypes.^126^ Accordingly, model outputs should not be limited to an uninterpretable risk score. It could be designed to estimate the probability of inflamed or immune‐excluded states, together with treatment‐response or resistance‐risk scores, thereby translating complex immune ecosystems into quantitative indicators for patient stratification.

Cross‐cancer studies have provided methodological support for this translational pathway. For example, in a multi‐omics study of pancreatic cancer, artificial neural network approaches were used to select candidate features from high‐dimensional transcriptomic data and construct classification models.^127^ Another study in hepatocellular carcinoma further demonstrated an approach for mapping routine pathological images to high‐dimensional immune ecosystems: the study extracted deep learning and morphological features from H&E whole‐slide images, associated them with transcriptomically defined NETs activity, and further integrated single‐cell and spatial transcriptomics to dissect the relevant immune cell populations and their spatial relationships. The resulting pathology‐based risk model retained predictive ability in an independently held‐out validation set.^48^ Although these studies were not conducted in OC, their main value lies in providing a transferable technical strategy: high‐dimensional omics are used to establish biologically defined reference states, while more clinically accessible data types are trained to recognize these states.

Building on this concept, a more specific AI‐assisted immune‐stratification framework could be developed for HGSOC. Single‐cell and spatial omics could first be used to define biologically meaningful immune states. Conventional H&E or multiplex IF images, *BRCA*/HRD status, and clinical data are then used as the main model inputs. Finally, the model outputs the patient's immune ecotype classification and the corresponding probability of benefit from immunotherapy. In addition, longitudinal data derived from repeat tissue biopsies, cfDNA, or ascites may be integrated during treatment to refine and update therapeutic strategies.

It should be noted that such models can only be translated from research tools into clinical decision support systems after rigorous validation. During model development, cross‐validation and independently held‐out test sets should be used to control overfitting, with feature selection, model training, and testing processes kept strictly separate. This should be followed by independent multicentre external validation across different hospitals, patient populations, and analytical platforms. AI models should subsequently undergo prospective validation to assess their calibration, stability and incremental predictive value over existing clinical indicators.

Therefore, the future development of AI in precision immunotherapy for OC should focus not on constructing increasingly complex algorithms, but on establishing a clear “multimodal input‐immune‐state identification‐treatment‐benefit prediction‐independent validation‐clinical decision‐making” pathway.

### 6.3 Future research priorities

Alongside efforts to standardize biomarkers and implement precision decision‐support tools, future OC research should further narrow the gap between mechanistic discovery and clinical application. Basic research should move beyond repeated descriptions of established immune‐cell populations and signalling pathways toward identifying the key biological events that govern early immune escape, peritoneal dissemination, and adaptive evolution under therapeutic pressure. Attention should be given to the dynamic interactions between tumour cells and immune, stromal, and tissue‐specific microenvironmental components, with the aim of identifying vulnerabilities that arise early during disease progression and remain therapeutically actionable.

At the same time, the evaluation of novel therapeutic targets should move beyond differential expression toward the demonstration of clear functional dependency and a defined therapeutic window. For OCs lacking conventional actionable drivers, or that have developed treatment resistance, non‐mutational dependencies, non‐coding RNAs, and microenvironment‐related vulnerabilities may provide additional therapeutic opportunities. Advances in novel delivery platforms, including liposomal nanoparticles and injectable nanocomposite hydrogels, have the potential to enhance drug exposure at target sites and reduce systemic toxicity.

**FIGURE 5:**
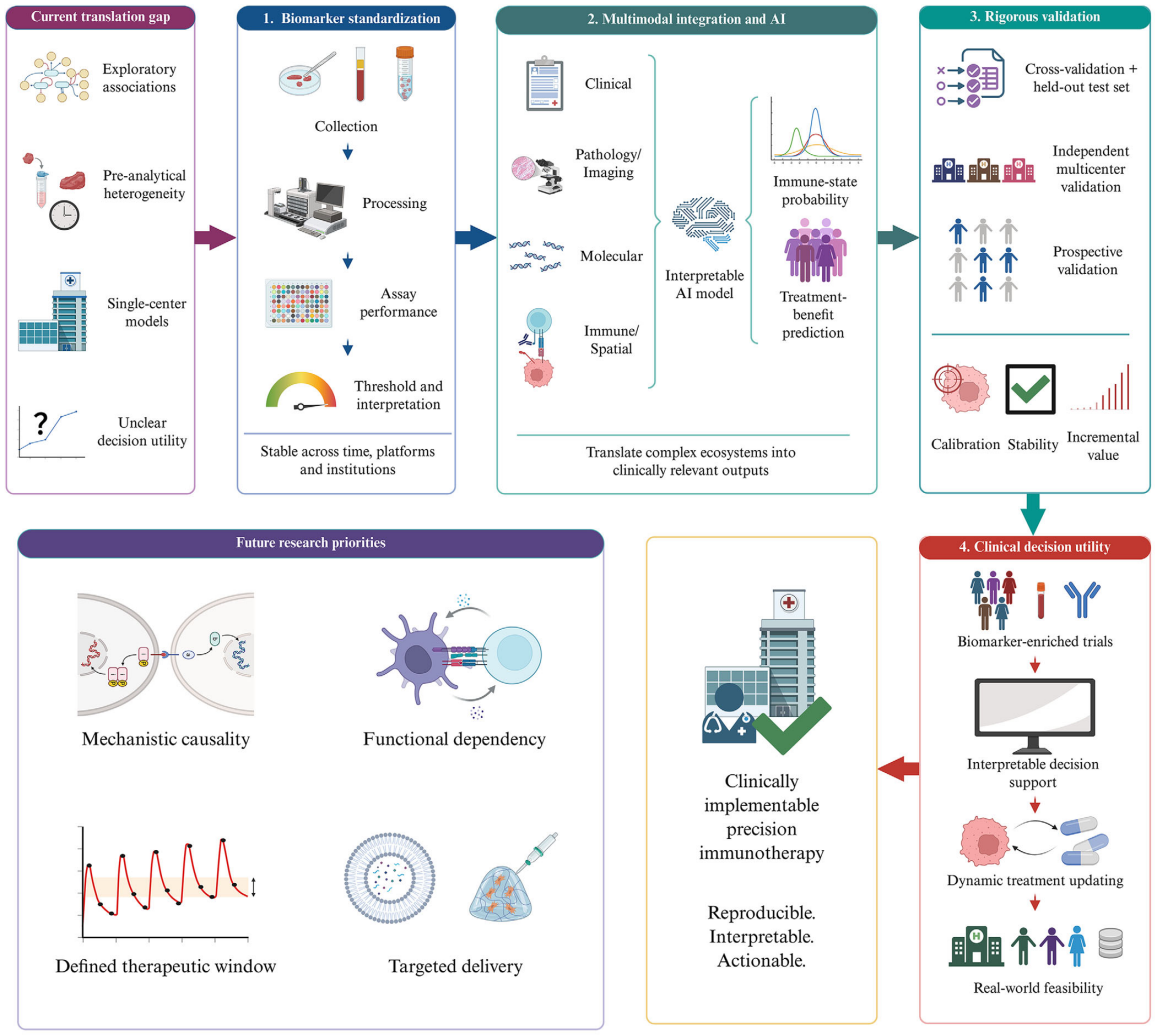
Clinical translation of precision immunotherapy. Successful translation requires standardized assays, multimodal and AI‐assisted integration, independent multicentre validation, prospective demonstration of clinical utility and feasible implementation.

Future research should integrate biological relevance, technical feasibility, and clinical need from the earliest stages of study design, facilitating the transition of precision immunotherapy from an expanding body of experimental discoveries to clinically testable and implementable strategies (Figure 5).

## 7 CONCLUSION AND PERSPECTIVES

Immunotherapy in OC has entered a stage where improving efficacy requires more than identifying additional therapeutic targets or combination regimens. The major challenge is to understand how heterogeneous and evolving immune ecosystems influence treatment response. By integrating spatial organization, temporal evolution and biomarker‐guided stratification, future immunotherapy strategies may move beyond generalized approaches toward more context‐specific interventions. A comprehensive summary of the clinical studies supporting the immunotherapeutic strategies discussed in this review, together with their corresponding levels of evidence, is provided in Table S1&S2.

A key priority for the field is to translate emerging biological insights into clinically applicable decision frameworks. This will require prospective validation of predictive biomarkers, rational design of biomarker‐enriched clinical trials and development of strategies that can adapt to changes in tumour immune states during treatment. Ultimately, the future of OC immunotherapy will depend on bridging mechanistic discovery with clinical implementation, enabling treatment decisions to be guided by the evolving biology of individual tumours rather than empirical approaches alone.

## AUTHOR CONTRIBUTIONS

Yanfang Gu, Shaojie Zhao, Bing Zhang and Yan Zhang conceptualized the review. Xinfeng Yang, Yida Wang, Haiyue You, Jingyi Gao, Yangkun Feng and Feng Zhang wrote the manuscript. Xinfeng Yang, Yida Wang, Haiyue You and Jingyi Gao prepared the figures and tables. Yanfang Gu, Shaojie Zhao, Bing Zhang and Yan Zhang critically reviewed and edited the manuscript. Yan Zhang and Shaojie Zhao got funding support. All authors read and approved the final manuscript.

## CONFLICT OF INTEREST STATEMENT

The authors declare no conflicts of interest.

## ETHICS STATEMENT

The authors have nothing to report.

## Supporting information



Supporting Information

## Data Availability

Data and materials can be provided upon reasonable request to the corresponding author.
