# Supplementary material for: Spatiotemporal immune heterogeneity and precision immunotherapy for ovarian cancer
Source: Clin Transl Med. 2026 Sep 14;16(9):e70836. doi: 10.1002/ctm2.70836 (PMC13574541; doi:10.1002/ctm2.70836)
Supplement: Supplementary file 1 [file CTM2-16-e70836-s001.docx]

**Table S1. Evidence grading system.**

| Grade | Evidence design |
| --- | --- |
| I | Prospective randomized phase III / regulatory evidence |
| I | Prospective randomized phase II |
| II | Prospective single-arm phase I/II |
| II | Prospective or paired longitudinal translational study |
| III | Multicenter/large pooled retrospective cohort or external validation |
| III | Single-center retrospective cohort or outcome correlation |
| IV | Exploratory human tissue single-cell/spatial/multi-omics |
| IV | Patient-derived ex vivo/in vitro functional evidence |
| V | Ovarian-cancer animal model |
| V | In vitro only, computational inference, or cross-cancer extrapolation |

**Table S2. Comprehensive evidence matrix.**

| References | Design classification | Evidence source | Grade |
| --- | --- | --- | --- |
| [1,3] | Population registry / descriptive epidemiology | Clinical samples/data | III-A |
| [8] | Prospective randomized phase III | Clinical samples/data | I-A |
| [9] | Prospective randomized phase III | Clinical samples/data | I-A |
| [10] | Prospective randomized phase III | Clinical samples/data | I-A |
| [12] | Single-center retrospective cohort | Clinical samples + single-cell/spatial omics | III-B |
| [14,15] | Single-center retrospective cohort | Clinical samples/data | III-B |
| [17,19] | Clinical-sample correlation or cross-sectional analysis | Clinical samples + functional assays | III-B |
| [16] | Exploratory human single-cell/spatial/multi-omics | Clinical samples + single-cell/spatial omics | IV-A |
| [18] | Clinical-sample correlation or cross-sectional analysis | Clinical samples/data | IV-A |
| [20,21] | Exploratory human single-cell/spatial/multi-omics | Clinical samples + single-cell/spatial omics | IV-A |
| [25] | Prospective/paired longitudinal translational study | Clinical samples + functional assays | II-B |
| [26] | Prospective/paired longitudinal translational study | Clinical samples/data | II-B |
| [27] | Prospective/paired longitudinal translational study | Clinical samples + single-cell/spatial omics + functional assays | II-B |
| [29] | Prospective/paired longitudinal translational study | Clinical samples/data | II-B |
| [30] | Prospective single-arm phase I/II | Clinical samples + functional assays | II-A |
| [31] | Multicenter/pooled retrospective cohort or external validation | Clinical samples + single-cell/spatial omics | III-A |
| [33] | Exploratory human single-cell/spatial/multi-omics | Clinical samples + single-cell/spatial omics | IV-A |
| [35] | Clinical-sample correlation or cross-sectional analysis | Clinical samples + single-cell/spatial omics | III-B |
| [36] | Patient-derived ex vivo/in vitro functional study | Clinical samples + functional assays | IV-B |
| [37] | Patient-derived ex vivo/in vitro functional study | Clinical samples + functional assays | IV-B |
| [38] | Single-center retrospective cohort | Clinical samples + functional assays | III-B |
| [39] | Exploratory human single-cell/spatial/multi-omics | Clinical samples + single-cell/spatial omics | IV-A |
| [41] | Clinical-sample correlation or cross-sectional analysis | Clinical samples/data | III-B |
| [22,42] | Multicenter/pooled retrospective cohort or external validation | Clinical samples/data | III-A |
| [42] | Patient-derived ex vivo/in vitro functional study | Clinical samples + functional assays | IV-B |
| [43] | Clinical-sample correlation or cross-sectional analysis | Clinical samples + animal model + functional assays | IV-A |
| [44] | Exploratory human single-cell/spatial/multi-omics | Clinical samples + single-cell/spatial omics + animal model | IV-A |
| [45] | Ovarian-cancer animal model | Animal model | V-A |
| [49] | Exploratory human single-cell/spatial/multi-omics | Clinical samples + single-cell/spatial omics + functional assays | IV-A |
| [50] | Exploratory human single-cell/spatial/multi-omics | Clinical samples + single-cell/spatial omics | IV-A |
| [51] | Patient-derived ex vivo/in vitro functional study | Clinical samples + functional assays | IV-B |
| [52] | Ovarian-cancer animal model | Animal model + in vitro functional assays | V-A |
| [53] | Patient-derived ex vivo/in vitro functional study | Clinical samples + functional assays | IV-B |
| [55] | Clinical-sample correlation or cross-sectional analysis | Clinical samples/data | III-B |
| [56] | Exploratory human single-cell/spatial/multi-omics | Clinical samples + single-cell/spatial omics | IV-A |
| [57] | Prospective randomized phase III | Clinical samples/data | I-A |
| [58] | Prospective randomized phase III | Clinical samples/data | I-A |
| [60] | Multicenter/pooled retrospective cohort or external validation | Clinical samples/data | III-A |
| [61] | Multicenter/pooled retrospective cohort or external validation | Clinical samples/data | III-A |
| [62] | Multicenter/pooled retrospective cohort or external validation | Clinical samples/data | III-A |
| [63] | Prospective single-arm phase I/II | Clinical samples/data | II-A |
| [64] | Multicenter/pooled retrospective cohort or external validation | Clinical samples + single-cell/spatial omics | III-A |
| [65] | Multicenter/pooled retrospective cohort or external validation | Clinical samples + single-cell/spatial omics | III-A |
| [66,68] | Clinical-sample correlation or cross-sectional analysis | Clinical samples/data | III-A |
| [7,67] | Prospective single-arm phase I/II | Clinical samples/data | II-A |
| [69] | Patient-derived ex vivo/in vitro functional study | Clinical samples + functional assays | IV-B |
| [70] | Clinical-sample correlation or cross-sectional analysis | Clinical samples/data | IV-A |
| [71] | Clinical-sample correlation or cross-sectional analysis | Clinical samples/data | IV-A |
| [72] | Clinical-sample correlation or cross-sectional analysis | Clinical samples/data | IV-A |
| [73] | Non-ovarian/cross-cancer or inferential evidence | Cross-cancer data / computational inference | V-B |
| [75] | Prospective/paired longitudinal translational study | Clinical samples/data | II-B |
| [76] | Prospective/paired longitudinal translational study | Clinical samples/data | II-B |
| [77,78] | Multicenter/pooled retrospective cohort or external validation | Clinical samples/data | III-A |
| [79] | Patient-derived ex vivo/in vitro functional study | Clinical samples + functional assays | IV-B |
| [81] | Prospective single-arm phase I/II | Clinical samples/data | II-A |
| [82] | Prospective single-arm phase I/II | Clinical samples/data | II-A |
| [88] | Prospective single-arm phase I/II | Clinical samples + functional assays | II-A |
| [97] | Prospective randomized phase III | Clinical samples/data | I-A |
| [101] | Prospective randomized phase II | Clinical samples/data | I-B |
| [102] | Prospective single-arm phase I/II | Clinical samples/data | II-A |
| [118] | Prospective randomized phase III | Clinical samples/data | I-A |
| [119] | Prospective randomized phase III | Clinical samples/data | I-A |
| [120] | Prospective randomized phase III | Clinical samples/data | I-A |
| [104,105] | Prospective randomized phase III | Clinical samples/data | I-A |
| [106] | Prospective randomized phase III | Clinical samples/data | I-A |
| [121] | Prospective randomized phase II | Clinical samples/data | I-B |
| [122] | Prospective randomized phase II | Clinical samples/data | I-B |
| [123] | Prospective single-arm phase I/II | Clinical samples/data | II-A |
| [103] | Patient-derived ex vivo/in vitro functional study | Clinical samples + functional assays | IV-B |
| [114] | Clinical-sample correlation or cross-sectional analysis | Clinical samples/data | III-B |
| [117] | Clinical-sample correlation or cross-sectional analysis | Clinical samples/data | III-B |
| [125] | Multicenter/pooled retrospective cohort or external validation | Clinical samples/data | III-A |
| [113] | Non-ovarian/cross-cancer or inferential evidence | Animal model + in vitro functional assays | V-B |
| [124] | Non-ovarian/cross-cancer or inferential evidence | Cross-cancer data / computational inference | V-B |
| [107] | Ovarian-cancer animal model | Animal model + in vitro functional assays | V-A |
| [108-110,116] | Ovarian-cancer animal model | Animal model + in vitro functional assays | V-A |
| [115] | Patient-derived ex vivo/in vitro functional study | Clinical samples + functional assays | IV-B |
| [48] | Non-ovarian/cross-cancer or inferential evidence | Cross-cancer data / computational inference | V-B |
| [127] | Non-ovarian/cross-cancer or inferential evidence | Cross-cancer data / computational inference | V-B |
